# Effects of Proline on Internal Friction in Simulated Folding Dynamics of Several Alanine-Based α-Helical Peptides

**DOI:** 10.1021/acs.jpcb.4c00623

**Published:** 2024-04-12

**Authors:** Adam Świątek, Krzysztof Kuczera, Robert Szoszkiewicz

**Affiliations:** † Faculty of Chemistry, Biological and Chemical Research Centre, University of Warsaw, Żwirki i Wigury 101, 02-089 Warsaw, Poland; ‡ Department of Chemistry, The University of Kansas, Lawrence, Kansas 66045, United States; § Department of Molecular Biosciences, The University of Kansas, Lawrence, Kansas 66045, United States

## Abstract

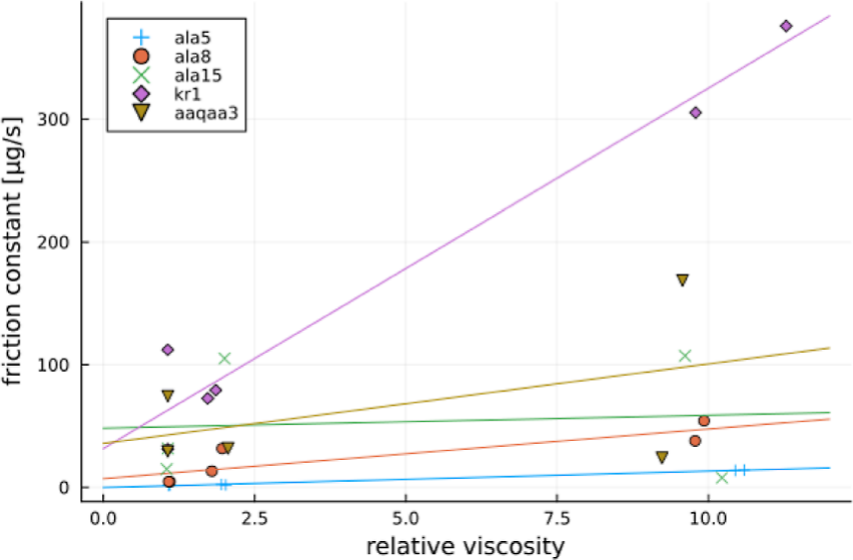

We have studied in silico the effect of proline, a model cosolvent, on local and global friction coefficients in (un)folding of several typical alanine-based α-helical peptides. Local friction is related to dwell times of a single, ensemble-averaged hydrogen bond (HB) within each peptide. Global friction is related to energy dissipated in a series of configurational changes of each peptide experienced by increasing the number of HBs during folding. Both of these approaches are important in relation to future atomic force microscopic-based measurements of internal friction via force-clamp single-molecule force spectroscopy. Molecular dynamics (MD) simulations for six peptides, namely, ALA5, ALA8, ALA15, ALA21, (AAQAA)_3_, and H_2_N–GN(AAQAA)_2_G–COONH_2_, have been conducted at 2 and 5 M proline solutions in water. Using previously obtained MD data for these peptides in pure water as well as upgraded theoretical models, we obtained variations of local and global internal friction coefficients as a function of solution viscosity. The results showed the substantial role of proline in stabilizing the folded state and slowing the overall folding dynamics. Consequently, larger friction coefficients were obtained at larger viscosities. The local and global internal friction, i.e., respective, friction coefficients approximated to zero viscosity, was also obtained. The evolution of friction coefficients with viscosity was weakly dependent on the number of concurrent folding pathways but was rather dominated by a stabilizing effect of proline on the folded states. Obtained values of local and global internal friction showed qualitatively similar results and a clear dependency on the structure of the studied peptide.

## Introduction

Despite being acclaimed as one of the big scientific questions for the next quarter century in 2005 by Science magazine,^1^ studies of protein folding still continue to occupy a modest place in modern science. Besides prominent advances in experimental methods already well-known in the previous decades, such as nuclear magnetic resonance,^2^ fast microfluidic-based stop-flow T-jump methods,^3^ and crystallographic methods, there are novel experimental methods, including cryo-electron microscopy methods^4,5^ and confined/microfluidic-enhanced Raman methods.^6^ In addition, novel molecular dynamics (MD)^7^ methods have been developed as well as entirely new computational approaches such as Alpha-Fold.^8^ Nevertheless, there is still room for additional proxies to probe structural changes during folding for large proteins as well as small peptides. In relation to peptides, minuscule structural changes occurring during their folding are too difficult to catch experimentally, but these - depending on the particular environment - are of paramount importance for various applications such as peptide-based delivery systems and peptide-based functional assemblies.^9,10^

One promising approach to studying minute structural changes of simple peptides is single-molecule force spectroscopy (SMFS), wherein the vibrational response of a single macromolecule is probed by manipulating the molecule by an atomic force microscopy (AFM) tip. SMFS–AFM applies forces within physiological range, which are registered by the same tip.^11,12^ An interesting idea within this approach is to measure the mechanical properties of the system under study and use these properties to infer information about structural changes. One concrete theoretical framework for doing so is an elaboration of the rheological Kelvin–Voigt model by Ploscariu et al.^13^ Therein, a protein molecule is abstracted by a system of molecular stiffness and friction coefficients. The friction coefficients represent energy dissipation, with a salient separation into solvent–polymer friction and internal friction arising from intrapolymer interactions.

Several studies, also experimental, of friction coefficients and internal friction for single peptides and proteins have already been conducted.^14−20^ The friction has been characterized in various ways. One possibility is to treat the polymer as a viscoelastic material and derive a viscoelastic friction coefficient in the units of kilograms per second while using models of damped harmonic oscillator and/or various incarnations of Langevin equations, such as Rouse models with internal friction (RIF).^16,19−23^ A different possibility is to instead focus on reaction rates and their associated characteristic times in units of seconds, usually through fits to, respective, autocorrelation functions. Both of these approaches in the framework of RIF models yield linear dependence of friction coefficients with viscosity and lead to *internal friction* at zero viscosity.^17,24−26^ Noteworthy, direct computational approaches to obtain internal friction, e.g., without the data of viscosity dependence of friction coefficients, have also appeared.^27^ Therein, internal friction has been calculated directly from autocorrelation functions of interatomic forces calculated over appropriate subsets of molecules.

Recently, we have proposed some models for extracting friction coefficients from MD simulations, which can be related to both approaches and are expected to relate to the SMFS–AFM studies as well.^28^ In particular, we defined *local friction coefficients* relating to reconfigurations of the mean ensemble-averaged hydrogen bond (HB) within each peptide. We also defined *global friction coefficients* related to energy dissipated due to overall conformational changes of each peptide experienced throughout its folding. From prospective SMFS–AFM experiments, both kinds of approaches are important. In the force-clamp AFM, a given tensile force is applied to a single protein or peptide molecule clamped between an arbitrary surface and an AFM tip. As the force gets larger, only a limited protein motion is expected within a stretched protein, so that at its limit, mere single HB switches become possible. Thus, FC-AFM measurements at substantial stretching forces would relate to the local friction coefficients. In contrast, integrating over all possible single HB switches and conducting the force-clamp AFM experiments at low clamping forces is expected to yield global friction coefficients. By performing the FC-AFM studies at various viscosities, the, respective, *local* and *global internal friction* can be obtained, which according to our knowledge has not been done yet both experimentally and computationally. Therefore, a need exists for finding *local* and *global internal friction* in the case of several model systems and discussing their origin.

Herein, we concentrate on finding out in silico the *local* and *global internal friction* in the case of several model alanine-based peptides known to fold into α-helical structures. We do so by introduction of a proline cosolvent, which allows for simulations at various viscosities and thus extrapolations to zero viscosity for estimates of, respective, internal friction. In addition, the effects of proline, which is a typical osmolyte, on the friction coefficients obtained at nonzero viscosities are discussed.

Due to the importance of the helix in biology, this structure has been the subject of a very large number of studies. A recent article on alanine helix folding simulations may be found in ref (29). An overview of recent simulations and comparison between modeling and experimental data has been presented in ref (30), while earlier studies are discussed in ref (31) and references therein. While osmolytes’ primary role in a cell is to regulate osmotic pressure, they also have important effects on protein folding, generally by encouraging formation of the folded state to oppose denaturation by urea or other factors.^32−34^ Their most generic action mechanism is to strengthen the usual hydrophobic interactions driving folding. Proline has been found to stabilize the folded state in this way but also to increase solvent viscosity. Nevertheless, its overall effect on protein stability is small compared to other osmolytes.^33^ In addition, the cosolvent tends to be excluded from the peptide backbone and may form specific interactions with the side chains.^35,36^ In the course of this work, we observed and discussed such effects. We also produced theoretical advances related to finding out the, respective, reconfigurational times associated with internal friction through dwell time analysis.^37,38^

## Materials and Methods

### MD Simulations

Three types of alanine-based peptides were considered in this work: (i) alanine-only peptides with 5, 8, 15, and 21 residues, (ii) the (AAQAA)_3_ peptide, and (iii) the KR1 peptide. The KR1 peptide had the following formula: H_2_N–GN(AAQAA)_2_G–COONH_2_. All peptides except KR1 were acetylated at their N-ends and amidated at their C-ends. The C-end amide termination was used in order to prevent the appearance of a charged acid group and increase the likelihood of HB formation.

Several MD simulations of these peptides were carried out as a part of this work in 2 and 5 M proline. The 2 and 5 M proline concentrations were chosen for two reasons. First, these are high enough concentrations that an observable difference of system properties from the 0 M conditions could be found. Second, at least three points, i.e., 0, 2, and 5 M, were desired for further calculations of internal friction, as presented later. The results for Ala21 in 5 M proline solution are not presented since the simulation would take too long to run to achieve desired statistics, i.e., with at least several folding/unfolding transitions. For all peptides, previous data for pure water solvent was used as a comparison.^28^

The simulations utilized the GROMACS 5.1.4 package with the CHARMM36m force field.^39^ The CHARMM36m force field was specifically modified compared to the previous CHARMM36 version, including an improved many-body CMAP correction for peptide backbone conformations as well as updated salt–link interactions. These changes allow the consistent modeling of both ordered and disordered protein states. The CHARMM36m potential for proteins, in combination with the TIP3P water model, has been tested on structural, thermodynamic, and kinetic data for model peptide and protein systems. Thus, it is a good choice for simulating peptide folding. By consistently using the same potential in simulations with and without proline, we expect to observe systematic effects of adding the cosolvent.

In each case, two differing initial peptide configurations were considered in order to have a control for whether the configuration space of the peptides was sufficiently sampled. If so, then both trajectories should show the same state densities. In the first case, an extended structure was used to start with, and in the second, an idealized α-helical structure was the starting point. The trajectories started from the helical configuration will be labeled with “h” and those from the extended configuration will be labeled with “e” throughout this work. The systems were solvated with TIP3P water molecules^40^ and proline. Each system was neutralized by the addition of Na^+^ and Cl^–^ ions to a final salt concentration of 0.15 M. Before starting the actual MD simulations, each system was briefly equilibrated first with harmonic restraints and then without restraints under NPT conditions of 1 bar and 300 K. The goal was to equilibrate the solvent and relax local interactions in the peptide, for which a brief simulation (1 ns) was considered to be sufficient.^30^

The main MD run was then performed in the NVT ensemble, with a temperature of 300 K for 20 μs except for Ala5, which was simulated for 10 μs. Trajectory snapshots were saved every 2 ps and the simulation ran with a 2 fs time step. Nonbonded cutoffs were 1.2 nm, and the PME method was used to account for long-range electrostatic interactions.

### RMSDH Analysis

A standard definition of the RMSDH (root-mean-square deviation from the ideal helix) has been used here as follows
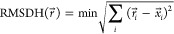
1where  are the actual positions of atoms and  are the positions of the same atoms in a configuration of an ideal helix. Therefore, in order to report a minimized RMSDH after each simulation step, the molecule has been realigned with the ideal helix via its translation and rotation.

RMSDH plots are the temporal evolution of the RMSDH data calculated per each simulation frame. The folding and unfolding events in the RMSDH plots correspond to major shifts in the RMSDH values. The RMSDH histograms showing the number of states at given RMSDH values have been interpreted as the equilibrium densities of the states. Consequently, the folded state naturally corresponds to RMSDH values in close vicinity to zero, while the unfolded states must comprise larger RMSDH values.

### Approach to Folding

Within this work, folding has been thermodynamically described as a simple two-stage process with the formal equation
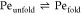
2where Pe_unfold_ denotes a peptide in the unfolded states and Pe_fold_ denotes a peptide in the folded α-helical state.

The folding equilibrium constant *K* for eq 2 reads
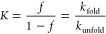
3where *f* is the fraction of the peptide in a folded state averaged over a whole trajectory, while *k*_fold_ and *k*_unfold_ are, respective, folding and unfolding reaction rate constants.

Following the refs (28) and (30) for helical peptides, the value of *f* is calculated from the fraction of the helical HBs formed throughout the simulation trajectory
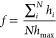
4where *h*_*i*_ is the number of helical HBs, i.e., the ones present in the folded state, within the *i*th frame out of *N* frames in total, and *h*_max_ is a maximum number of HBs in a folded state. We use a purely distance-based criterion for HB finding, in which an α-helical HB in residue *i* is formed when the distance *O*_*i*_...*N*_*i*+4_ is below 0.36 nm, with *O*_*i*_ the peptide carbonyl oxygen of residue *i* and *N*_*i*+4_ the peptide nitrogen of residue *i* + 4. In our experience, this method of helix content calculation yields similar results to analyzing Ramachandran maps or using the DSSP algorithm.^30^

The time series of *h*_*i*_ values were also used to calculate the reaction rate constants *k* according to^30^
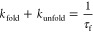
5where τ_f_ is the time constant obtained from the *h*_*i*_ autocorrelation function, fitted using a mono-exponential decay plus a constant (if needed). The autocorrelation function was defined in the usual way, as given in ref (28).

### Approach to Internal Friction

The *local friction coefficient*—pertaining to lasting of a single HB—has been calculated from the fluctuation–dissipation theorem as^28^
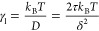
6where *D* is the diffusion coefficient obtained from 1-D Brownian motion as
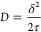
7

In eq 7, the value of δ is the mean elongation/shortening distance of a helix after addition/subtraction of one HB and τ is the dwell time obtained from the *h*_*i*_ time series. For more information about τ, see the section “dwell times” in the supplement.

The *global friction coefficient*—pertaining to an energy dissipation during an overall folding reaction for each peptide—has been calculated based on the work of Khatri et al.^23^ Using the model derived by Wosztyl et al.,^28^ the *global friction coefficient* is
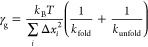
8where Δ*x*_*i*_ is the ensemble-averaged change in the end-to-end distance of the peptide after passing from *i* to *i* + 1 HBs along its folding trajectory. More information about calculations of Δ*x*_*i*_ will be presented in subsequent sections.

The values of *local* and *global friction coefficients* yield the values of *local* and *global internal friction* while approximated to zero viscosity according to previously formulated approaches in the field.^26,41^

Because of differences obtained between “e” and “h” trajectories, most calculations (except where noted otherwise) were performed separately for each trajectory, and where a value of friction coefficients (and other related variables) for a particular peptide/proline combination was needed, the values from the two trajectories were averaged and half of their difference was taken as the error. For this reason, all the used data were calculated separately for each trajectory (“e” or “h”) as well as the averaged data is appended to the Supporting Information.

### Water Diffusion Constants

Water diffusion constants were calculated from the simulation trajectory by using Gromacs gmx-msd program. It calculates an ensemble average mean square displacement (MSD) defined per-molecule as
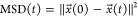
where  is the position of the molecule at time *t* treated as center of mass. Herein, the MSD values were calculated separately for chunks of the trajectory totaling 200 ns each for 0 and 5 M proline and 100 ns each for 2 M proline. In each case, only 10% of the total trajectory data were used. The chunks were then averaged.

The resulting average MSD is a linear function of time (after a nonlinear domain at short times), and its slope equals 6 times the diffusion constant, as can be derived by assuming 3D random motion for the particles and solving an appropriate differential equation for a Brownian diffusion.^42^

## Results and Discussion

Below, we will briefly present the MD results obtained for our studied peptides at various concentrations of proline cosolvent. Afterward, the MD results are used to derive estimates for the internal friction of these peptides.

### Visualizing the Reaction Coordinate’s Evolution at Various Proline Concentrations

**Figure 1:**
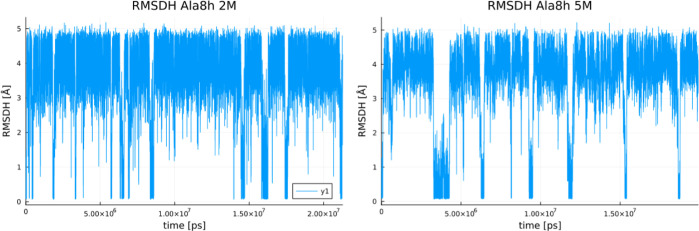
Comparison of RMSDH(t) between Ala8 in 2 M (left) and 5 M (right) proline for the simulations starting from the helical Ala8 conformation.

To start with, a typical thermodynamic analysis of the folding process has been conducted as previously described.^28,30^ We compare and discuss RMSDH values in MD trajectories at different proline concentrations since this parameter is commonly used as a proxy for folding. Obtained RMSDH trajectories for an ALA8 peptide at 2 and 5 M proline concentrations are shown in Figure 1. The same plots for all other peptides have been inserted into the Supporting Information.

Analyzing the RMSDH(t) plots qualitatively, we notice that with higher proline concentration, transitions occur more rarely, implying slower dynamics. A related quantitative approach is to obtain autocorrelation times from the RMSDH(t) plots and later calculate the forward and backward folding reaction rates using eqs 3 and 5. This has been done here, and the resulting values of *k*_fold_, *k*_unfold_, and *K* are presented in Table 1. Table 1 shows a decrease in both forward and backward reaction rates with increasing proline concentration. Slower dynamics might be related to crowding and overall higher viscosity induced by proline. In contrast, however, an equilibrium constant *K* increases with more proline present, which implies proline’s stabilizing effect on the folded state.

**Table 1:** Reaction Rates and Implied Equilibrium Constants for the Folding Reaction (Eq 2)^a^

peptide	proline conc.	*k*_unfold_ [1/μs]	*k*_fold_ [1/μs]	*K*
Ala5	5 M	30.0 ± 7.8	1.77 ± 0.01	0.0590 ± 0.0157
Ala8		3.82 ± 1.78	0.639 ± 0.132	0.168 ± 0.113
Ala15		0.623 ± 0.352	2.40 ± 1.11	3.86 ± 3.96
AAQAA3		0.910 ± 0.530	0.836 ± 0.476	0.92 ± 1.06
KR1		0.497 ± 0.200	0.141 ± 0.025	0.284 ± 0.164
Ala5	2 M	330 ± 4	11.3 ± 1.2	0.0343 ± 0.0041
Ala8		9.76 ± 3.27	1.08 ± 0.34	0.111 ± 0.072
Ala15		0.702 ± 0.063	0.637 ± 0.240	0.908 ± 0.423
AAQAA3		3.65 ± 0.09	1.19 ± 0.01	0.326 ± 0.011
KR1		4.00 ± 1.04	0.513 ± 0.033	0.128 ± 0.042
Ala5	0 M	443 ± 8	13.1 ± 1.0	0.0297 ± 0.0028
Ala8		74.5 ± 10.2	5.00 ± 0.39	0.0671 ± 0.0144
Ala15		11.7 ± 5.1	4.15 ± 2.31	0.356 ± 0.352
AAQAA3		6.85 ± 0.31	1.89 ± 0.12	0.276 ± 0.030
KR1		22.6 ± 4.9	1.31 ± 0.14	0.0581 ± 0.0188
Ala21		1.16 ± 0.18	1.66 ± 0.27	1.43 ± 0.45

a The errors of *k*_fold_ and *k*_unfold_ come from maximum differences between “e” and “h” trajectories under each conditions. The error of *K* has been obtained as the maximum error via a complete differential rule. Three significant digits and/or same accuracy for errors associated to each value.

To elaborate on the effects of proline on folding, the so-called 3D heat maps plotting the free energy as a function of the HB number and end-to-end distance were obtained. The free energy is calculated as *G* = −*RT*ln Ω, where *R* is the ideal gas constant, *T* is absolute temperature, and Ω refers to the number of conformations within a given bin, i.e., for a fixed HB number and within a given range of end-to-end distances. Heat maps defined in this way provide an easy means to understand representations of the folding funnel.^28,30^

**Figure 2:**
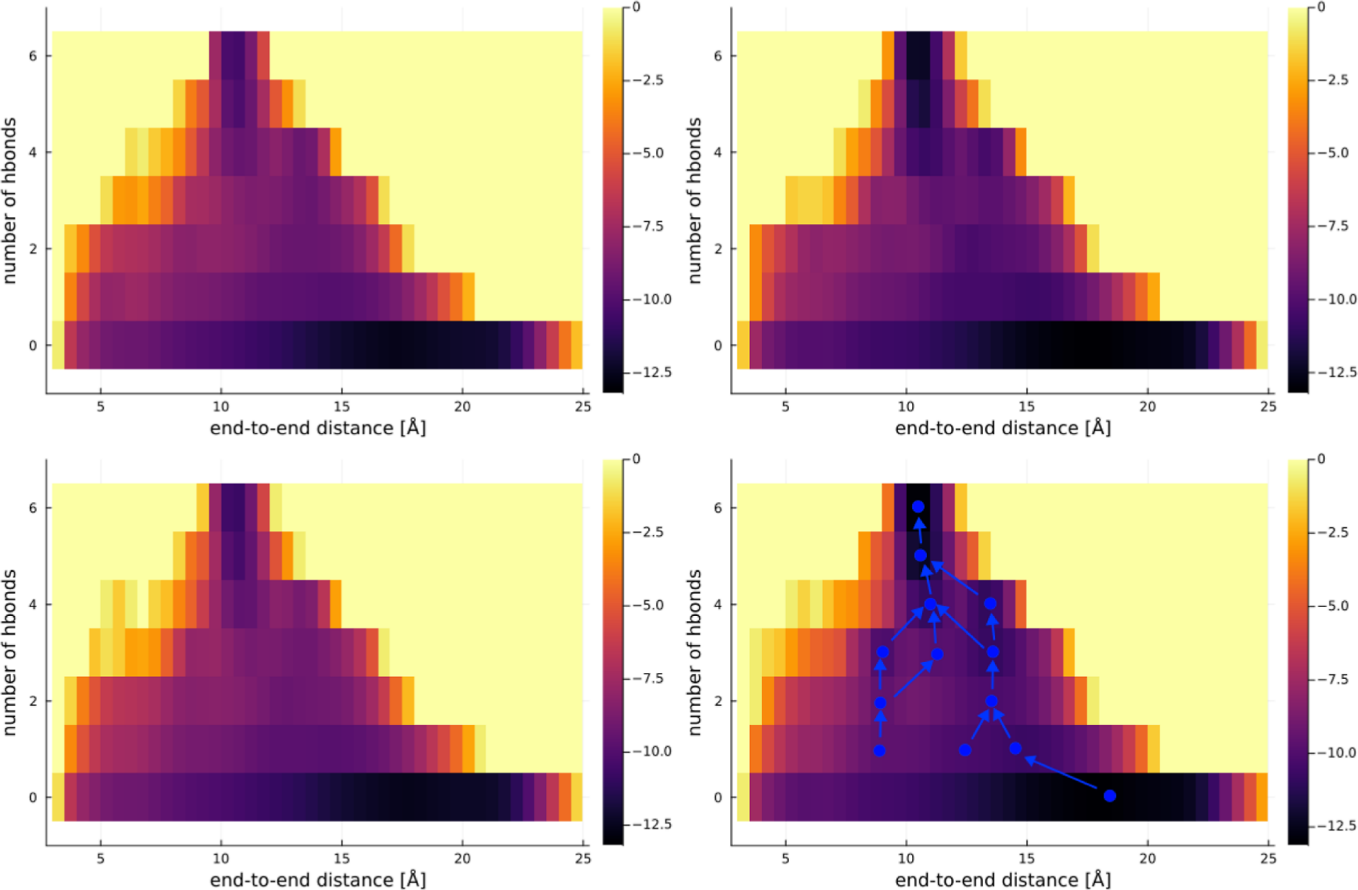
Representation of the free energy surface for ALA8. The top row is “h” configurations, and the bottom row “e”. The left is in pure water, and the right in 5 M proline. In the case of ALA8 “e” in 5 M proline, a detailed analysis of transitions between various populations characterized by similar end-to-end distances at given HBs yielded a map of folding pathways marked by blue arrows and lines.

**Figure 3:**
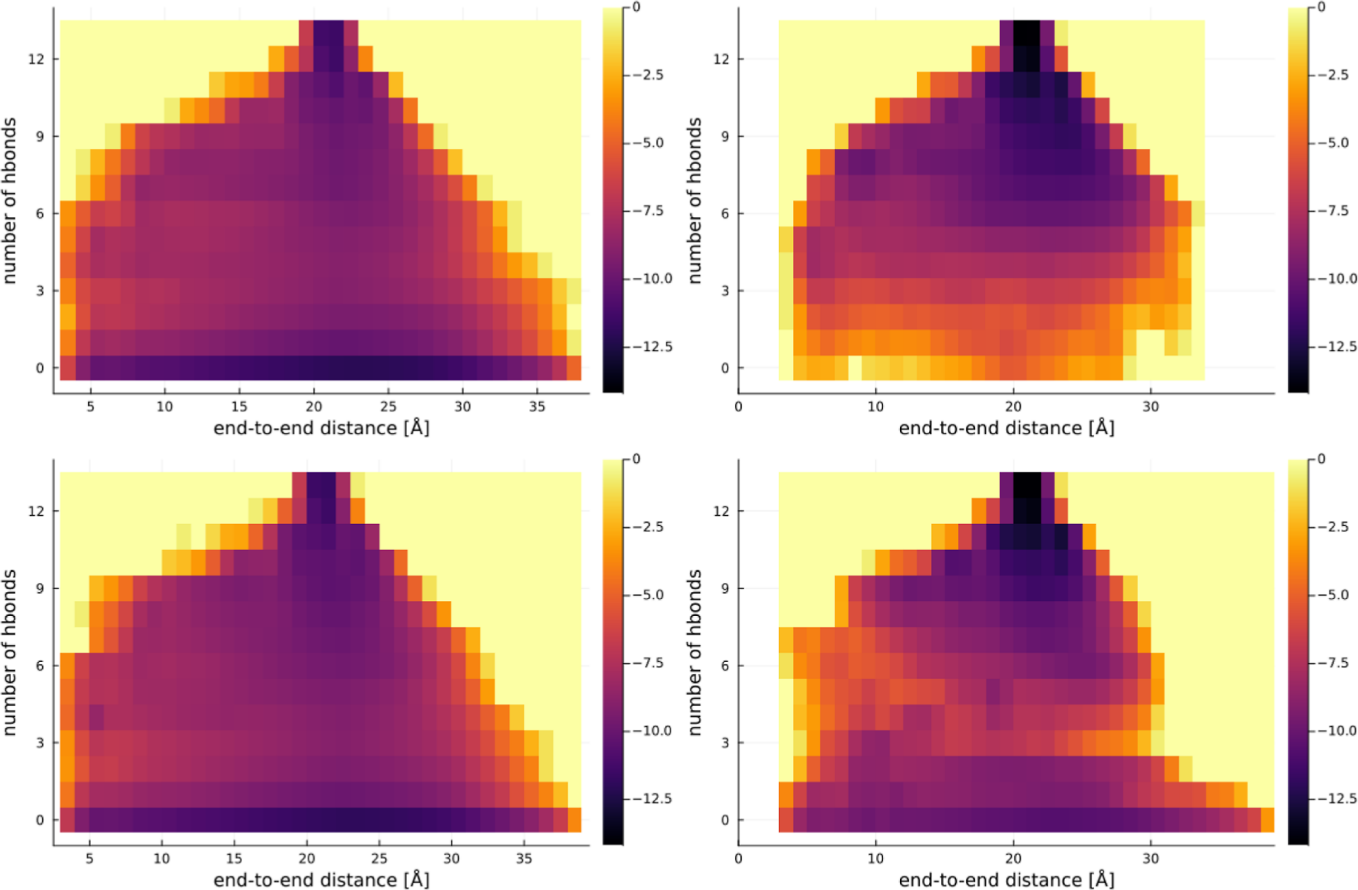
Representation of the free energy surface for ALA15. The top row is “h” configurations, and the bottom row “e”. The left is in pure water, and the right in 5 M proline.

**Figure 4:**
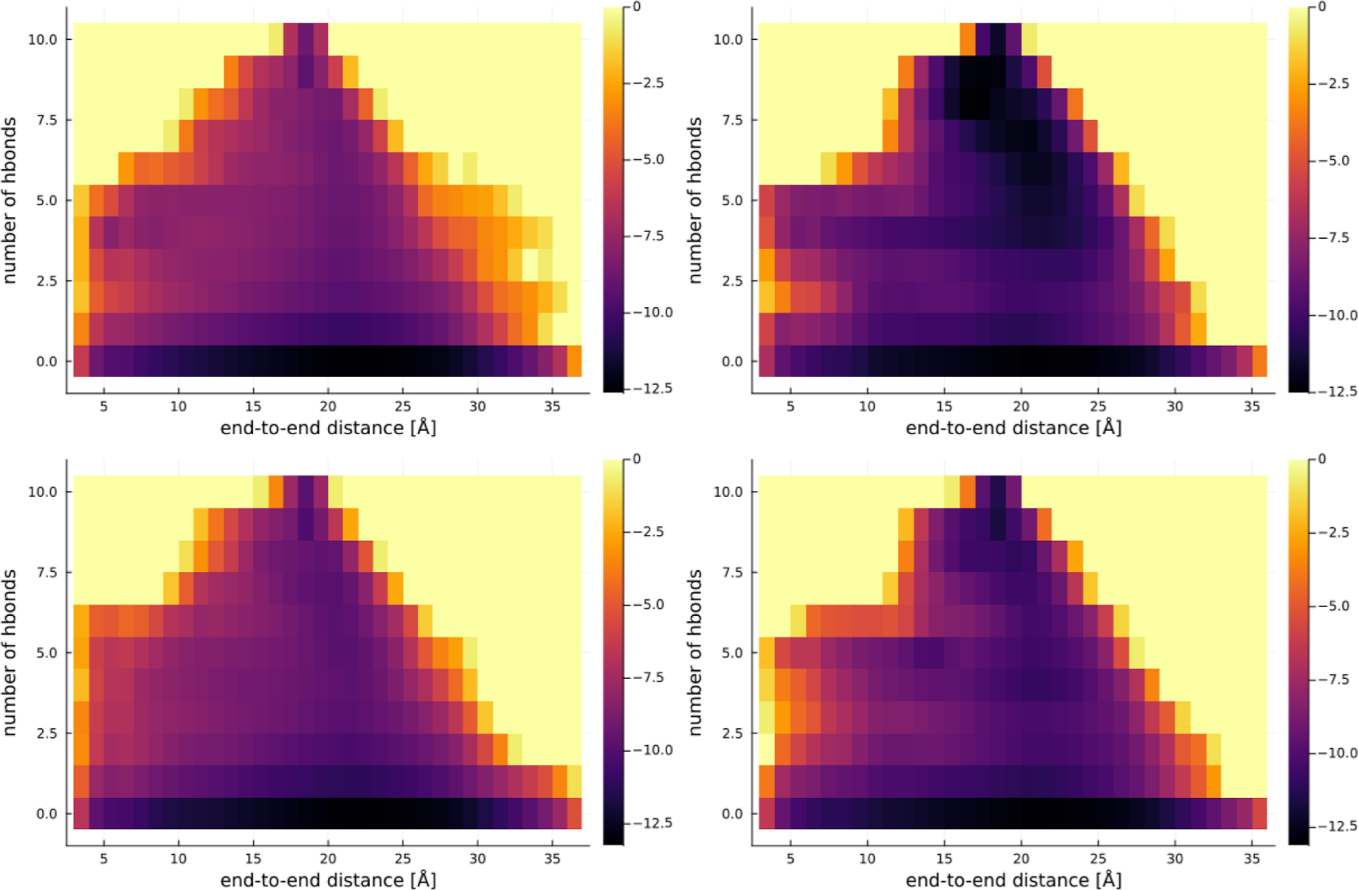
Representation of the free energy surface for KR1. The top row is “h” configurations, and the bottom row “e”. The left is in pure water, and the right in 5 M proline.

In Figures 2–4, we present and discuss heat maps for three representative examples, i.e., ALA8, ALA15, and KR1 peptides, in relation to proline concentration. For all cases, the top row in these figures relates to simulations starting from “h” configurations and the bottom row to “e” configurations. The left column is in pure water, and it was obtained from the data in ref (28) The right column is in 5 M proline. Figures of this sort for all peptides studied here have been included in the Supporting Information.

Already in the case of ALA8 in Figure 2, while comparing the left and right columns for water vs 5 M proline, respectively, one can observe that additional proline stabilizes the folded state, which results in lower free energy (darker colors) toward the top of the graph (highest HB number). However, the energy of the random coil state (at 0 HBs in the bottom of the graph) does not seem affected by proline. Furthermore, there is no notable effect of increasing proline concentration for the intermediate states, i.e., the ones with 1–4 HBs.

The stabilizing effect of proline on the folded state is also well visible for all other peptides; see the Supporting Information. Furthermore, for longer peptides, such as (AAQAA)_3_ and KR1 peptides, 5 M proline stabilizes not only the folded state but also partially folded states. In the case of the (AAQAA)_3_ peptide, the stabilized intermediate states concern configurations, which are fully folded at the ends and partially bent in the middle. In the case of KR1 peptide, it is the contrary, since additional partially folded states stabilized by proline are the ones with a helical region usually in a central portion of the peptide. Thus, glycines at the ends of the KR1 peptide do not help in producing stable partially folded configurations.^28^ Comparing all longer than eight aa peptides, there is no systematic trend in either central or terminal partially folded configurations stabilized by proline in the case of the partially folded peptides. Nevertheless, the basin of the partially folded states stabilized by proline is asymmetric; i.e., in the case of a lower number of HBs, longer end-to-end distances are more stable. An explanation for such behavior pertains to the properties of the helix itself, since elongating the helix straightens and shortens the peptide, and apparently bulky proline molecules further help this tendency. Overall, the stabilizing effect of proline in the case of partially folded configurations is rather nonspecific, i.e., it does not involve any particular HB pattern nor any new kinds of interactions.

Further effects of proline are manifested in slowing the folding dynamics. Interestingly, in the case of ALA8, a similar equilibrium distribution of states was reached by trajectories starting from the folded (“h”) and extended (“e”) states. This also underlines that the MD simulations for ALA8 were conducted for a sufficiently long time. However, ALA8 is the longest peptide for which this is true, as can be seen from the comparison for ALA15 in Figure 3. While for 2 M proline (not pictured here) and 0 M proline, the simulation length is sufficient, the comparison with 5 M proline shows for the “h” trajectory a clear tendency for exploring mostly more folded states near the top of the graph, and for “e” a similar bias, but for the less-folded states near the bottom. The “h” trajectory for ALA15 also contains undersampled states at 0 or 1 HB, seemingly having difficulty crossing the potential barrier at 3 HBs. The graphs for KR1 in Figure 4 also show slowdown of the folding dynamics in the case of 5 M proline, mostly manifesting in the deep energy “valley” to the center and top of the graph in the “h” trajectory, which is not present for the “e” trajectory. Interestingly, KR1 peptide shows a shallow local minimum at a 15 Å end-to-end distance and 4 HBs, with the valley of this minimum extending into low end-to-end distances of 7.5 Å or so. Therefore, competition between the helical state and this partially folded state might partially account for the low helical propensity of this peptide compared to its analogues. Summing it up, 5 M proline in the case of peptides longer than eight residues introduces some bias for exploring the states further from the original starting conditions. Similar bias appeared in both kinds of simulations, i.e., the ones starting from “e” and “h” configurations. Therefore, their mean value, besides carrying a large error, should not be affected.

Finally, upon further analysis, we observed that the populations of all individual helical HBs increase upon proline addition. This effect can be explained by changing solute–solvent interactions, and in more detail, exclusion of osmolytes from the peptide backbone, presence of specific proline-side chain interactions, and modification of water structure.^34−36^

### Internal Local Friction Estimates

To estimate the friction coefficients, we used two approaches. In the first one, which we call the *local approach*, we consider 1-D Brownian motion abstracted as elongation or shortening of a helix by one HB. This leads to an internal friction obtained from the Einstein fluctuation–dissipation theorem via eq 6 with a corresponding diffusion coefficient, *D*, defined in a standard way; see eq 7. A similar approach has already been applied in ref (28) for the case of simple peptides folding in pure water. In the current work, we present some novel strategies to obtain the critical variables in eq 6, i.e., δ and τ.

Concerning δ, the easiest method would be to consider a vertical rise per residue of an α-helix of 0.15 nm. However, some other methods could also be used. For example, one could consider the following situation. We calculate from MD runs a distance between a given O–N pair requiring that one of its neighboring O–N pairs has already formed a HB. Then, from such a distance, *d*, one subtracts a mean HB distance *d*_HB_. By doing so, one obtains a mean change in helix length associated with an additional HB formed in a cooperative manner. Using a detailed analysis described in the Supporting Information for all peptides at various proline concentrations, we obtained a corresponding δ = 0.34 ± 0.04 nm.

On one hand, the helix rise per residue tells how much a helix extends after the formation of an additional HB. On the other hand, our novel approach relates rather to the distance by which the residues must travel to cooperatively form a HB, i.e., while there is already one in the neighboring pair. Very interestingly, both of these approaches yield a fairly constant value of δ, which suggests that this property is indeed controlled by the backbone and does not depend on the proline concentration. Thus, to make our approach straightforward for other sets of data, we decide to follow up with a helix rise per residue, like in the previous works.^28^ However, if someone would like to use the other aforementioned approach to calculate δ, then it would be necessary to divide the local friction by a constant factor of 5.14 arising from (3.4/1.5)^2^.

Within our approach, the times τ correspond to the wait times necessary for extension or contraction of a helix due to formation or rupture of a single HB. Previously, they have been obtained as the shorter times from biexponential fits of RMSDH autocorrelation functions (ACFs).^28,30^ However, since our approach to internal friction dwells on HB evolution, using RMSDH(t) is like using an additional not directly related reaction coordinate. Noteworthy, the unfolded state from RMSDH(t) is not as well-defined as the folded one and may collect several very different states under one umbrella. It is also unclear why the state spatially furthest from the folded state in the RMSDH sense should be considered the “most unfolded”. Furthermore, the RMSDH analysis yields the times τ with large uncertainties due to high amplitude noise in RMSD ACFs and low fitting weights for the shorter times. These and other related problems are exemplified and described in detail in the Supporting Information. Consequently, we conclude that the RMSDH analysis does not yield the most relevant times τ and does not do it accurately enough.

Therefore, a different estimate for τ is desirable, and such can be sought from an analysis of the HB data directly. To this end, a dwell time approach has been used,^37^ which we describe in detail in the Supporting Information. The dwell times here correspond to the lifetimes of the single HBs. Out of measurements of such dwell times for each given studied peptide and using a particular HB within such a peptide, one obtains histograms of dwell time distributions. Using logarithmic dwell time histograms for better visualization of the obtained data,^37,38^ we were able to discern the times τ from inverses of the fitted decay constants. We started from single dwell time fits, i.e., monoexponential fits of the histogram generating functions, but these were not sufficient even in the case of the simplest structures, i.e., ALA5 and ALA8. In these cases, biexponential fits were used, which matched the data almost perfectly. Such great agreements gave us confidence to use the biexponential fitting approach in all other cases. Consequently, dwell times obtained for all the studied systems are listed in Table 2 together with the resulting values of the local friction obtained from eq 6.

**Table 2:** Average Time Needed to Change the Number of Helical Hydrogen Bonds in the Structure and the Corresponding Values of Local Internal Friction

peptide	proline	dwell time [ps]	local friction coefficient [ng/s]
Ala5	5 M	36.6 ± 0.7	13.5 ± 2.6
Ala8		81.4 ± 6.3	29.9 ± 2.3
Ala15		309 ± 12	114 ± 4
KR1		73.2 ± 1.6	26.9 ± 0.6
AAQAA3		61.6 ± 21.2	22.7 ± 7.8
Ala5	2 M	17.0 ± 0.1	6.25 ± 0.02
Ala8		41.5 ± 0.5	15.3 ± 0.2
Ala15		135 ± 1	49.9 ± 0.1
KR1		44.5 ± 4.6	16.4 ± 1.7
AAQAA3		63.8 ± 2.6	23.5 ± 1.0
Ala5	0 M	12.8 ± 0.1	4.71 ± 0.01
Ala8		28.3 ± 0.2	10.4 ± 0.1
Ala15		86.5 ± 1.5	31.8 ± 0.6
Ala21		109 ± 2	40.3 ± 0.6
AAQAA3		27.5 ± 0.1	10.1 ± 0.1

While inspecting the values of local friction coefficients for each given peptide, one can quickly notice that they increase with proline concentration. This can be understood through an increase of the times τ with proline concentration. There are two hypotheses that account for that. First one relates to a more restricted backbone motion at higher proline concentration. Second one relates to stabilization of each HB by proline molecules.

**Figure 5:**
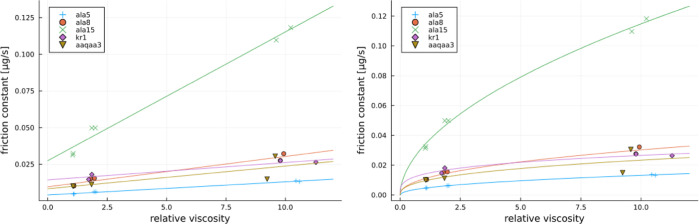
Local friction estimate as a function of relative viscosity for a variety of studied cases. On the left with a linear fit and on the right a power law fit.

In order to shed more light on this conundrum, in the next step, we embark on calculations of viscosities for solutions with various proline concentrations so as to estimate internal friction, which is the friction coefficient at zero viscosity, as previously described. In order to obtain viscosity, the values of water diffusion constant, *D*_w_, have to be calculated first. Our obtained values of *D*_w_ are roughly twice as much as their experimentally observed counterparts.^43^ The same behavior was also observed in another work using the TIP3P water model.^44^ Since the relative viscosity values are typically used to avoid experimental problems as well as to cancel dependence on the hydrodynamic radius, we also follow such approaches^41^ and report the ratio of water viscosity η at particular proline concentration to pure TIP3P water viscosity η_0_. Then, through typical relationships between viscosity and diffusion constants, such as Stokes models, the ratio of η/η_0_ is expected to be equal to the ratio of *D*_w0_/*D*_w_, where *D*_w0_ = 5.6635 × 10^–5^ cm^2^/s is the diffusion constant obtained for pure TIP3P water. Consequently, the plots of local friction against relative viscosity η/η_0_ can be obtained and these are presented in Figure 5.

To start discussing the relationships of local friction coefficients with viscosity, we first fit them with two previously utilized approaches.^41^ First, a linear relationship is fitted between local friction coefficients and viscosity according to the equation
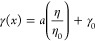
9with *a* being a slope and γ_0_ being an intercept with the meaning of internal friction.

At least some data, particularly for Ala15, appear better fitted with the power law as
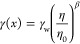
10with γ_w_ and β being the free parameters and γ_w_ having the meaning of friction coefficient in pure water. The obtained fit parameters are listed in Table 3 both for linear and power law fits.

**Table 3:** Fit Coefficients for Each Peptide on All Available data. γ_0_ is the Internal Friction Estimate

peptide	*a* [ng/s]	local internal friction, γ_0_ [ng/s]	γ_w_ [ng/s]	β
Ala5	0.897	4.09	4.54	0.462
Ala8	2.07	9.69	10.8	0.445
Ala15	8.78	27.5	33.3	0.538
KR1	1.18	14.4	14.0	0.278
AAQAA3	1.58	7.99	8.25	0.455

The power law approach, despite yielding no internal friction, has been proposed and utilized by previous works^41^ to explain faster dynamics of the system at higher viscosities than predicted by supposing a linear dependence produced by eq 9. While it is not related to internal friction, this interesting behavior could be explained by the breakdown of the Stokes law, supposing that various relaxation time scales are proportional to an inverse of the diffusion constant. However, relaxation times considered as the ones contributing to protein friction at large viscosities do not necessarily mean the times associated with peptide diffusion and do not necessarily lead to any breakdown of the Stokes law. As explained within ref (41), these are rather associated with not-fast-enough motion of the solvent molecules generally termed by solvent memory effects. Such memory effects manifest themselves at sufficiently large viscosities at similar time scales as protein relaxation times. Consequently, they provide for additional “lubrication” accelerating the protein motion via motion of solvent molecules. Herein, notably faster system dynamics at large viscosities than that expected from the linear relationship between friction constant and relative viscosity has been observed only in the case of one peptide, ALA15, so that linear approximations hold sufficiently well. This result is expected since local friction coefficients calculated here pertain to ensemble-averaged single-HB formation/rupture events, which in the case of an α helix are associated with very local motion of only several atoms involved.

Observing the results in Table 3, similar values of the parameter β are obtained as in the case of other simple α-helical proteins already studied in the literature,^41^ i.e., between 0.4 and 0.6 (except of KR1). This points to similar internal friction mechanisms in various ala-based alpha helical proteins.

Next, one can quickly notice that the values of γ_0_ and γ_w_ are very similar. Indeed, friction coefficients in pure water are enough to estimate internal friction without the need to obtain its relative viscosity dependence. In our most varying case of ALA15, we obtain 17% difference between its friction coefficient in water vs its internal friction, while in all other cases, it is less than 10%. Such a conclusion might apply only to the peptides studied here but shall certainly validate our earlier assumptions.^28^

**Figure 6:**
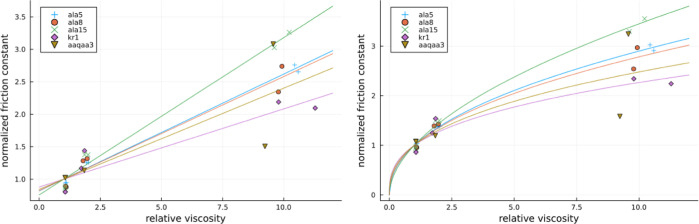
Normalized local friction estimate as a function of relative viscosity for a variety of studied cases. On the left with a linear fit and on the right a power law fit.

Finally, the values of local internal friction range between 4 and 28 ng/s. These will be later compared with the relevant literature. In order to observe more subtle differences between studied proteins and the effect of proline on friction coefficients, in Figure 6, we present the plots of their normalized local friction coefficients against relative viscosity. Normalization was performed by dividing all local friction values by their corresponding values obtained at a relative viscosity of one.

Observing the normalized plots, we can quickly notice substantial scattering of the normalized friction coefficient obtained for the AAQAA3 peptide in the case of extended and helical starting conformations in 5 M proline. These originated from particularly poor fits to the corresponding logarithmic dwell time histograms; see the Supporting Information. Nevertheless, the normalized local friction plots nicely show several dependencies of friction coefficients. ALA5 and ALA8 being the simplest peptides show similar growth rates of the local friction coefficients. ALA15 shows substantially faster growth of the friction coefficients, likely due to cooperativity and stabilizing effects for single HBs, which originate from other formed HBs and are strengthened by proline. These effects produce larger dwell times and are also reflected in higher *K* value for this peptide, see Table 1, compared with ALA5 and ALA8. Furthermore, KR1 and AAQAA3 contain some non-ALA residues, which in their case lead to slower growth of friction coefficients in comparison to ALA15, which has a similar number of residues. In this particular case, we observed lower free energy barriers for folding, smaller dwell times, and lower *K* values, which point toward a tendency of these peptides to stay within unfolded configuration rather than having difficulty to fold.

Overall, different structures among the same family of α-helical peptides result in different local internal friction and different growth mechanisms of friction coefficients with viscosity. Taken together, this is a desired indicator for future experimental discrimination of various α-helical structures through a local friction coefficient at various and particularly larger concentrations of osmolytes.

### Internal Global Friction Estimates

**Figure 7:**
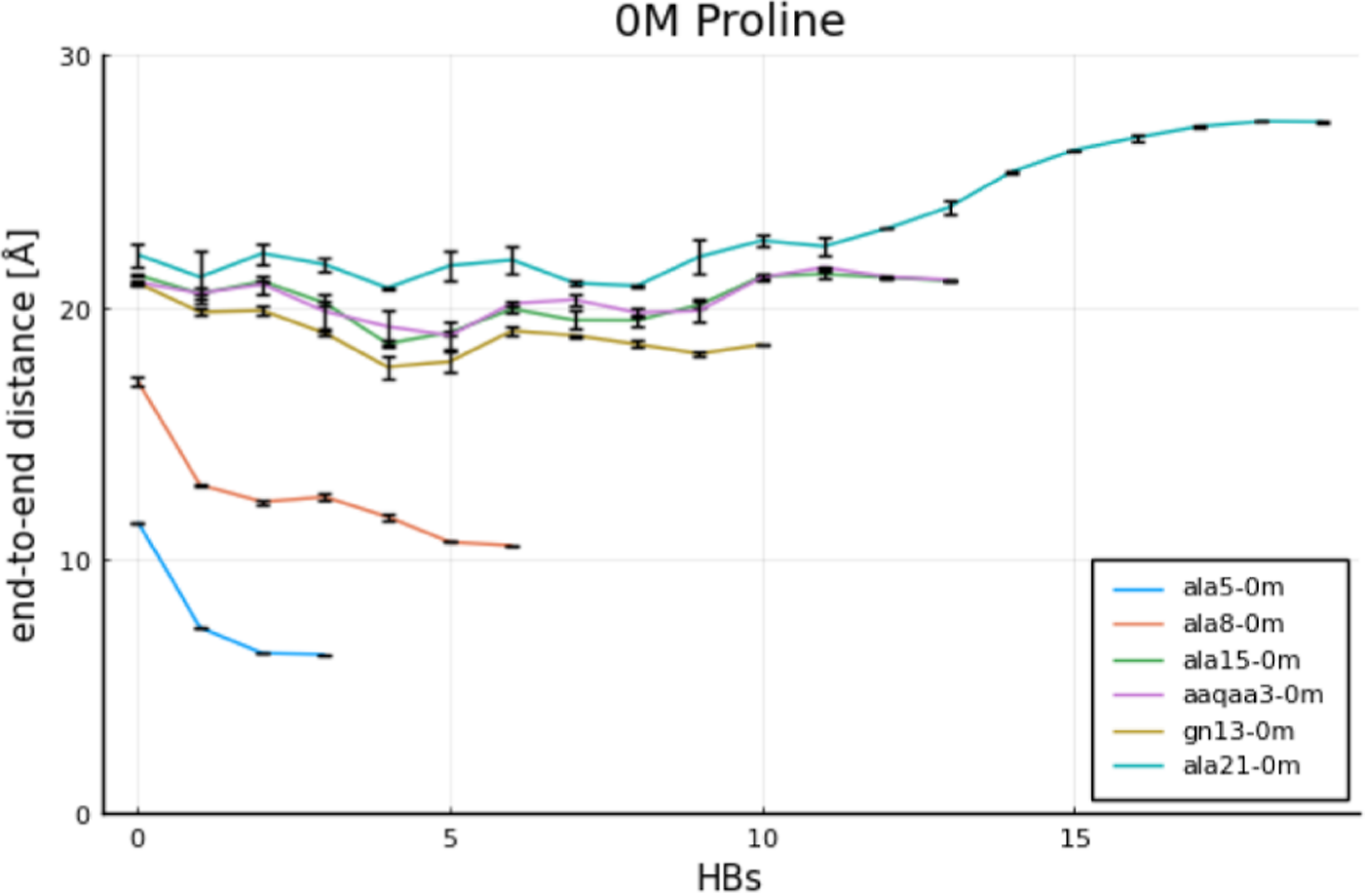
Movement of the center of mass *x*_*i*_ of the end-to-end distance histogram with the changing number of HBs for no proline.

**Figure 8:**
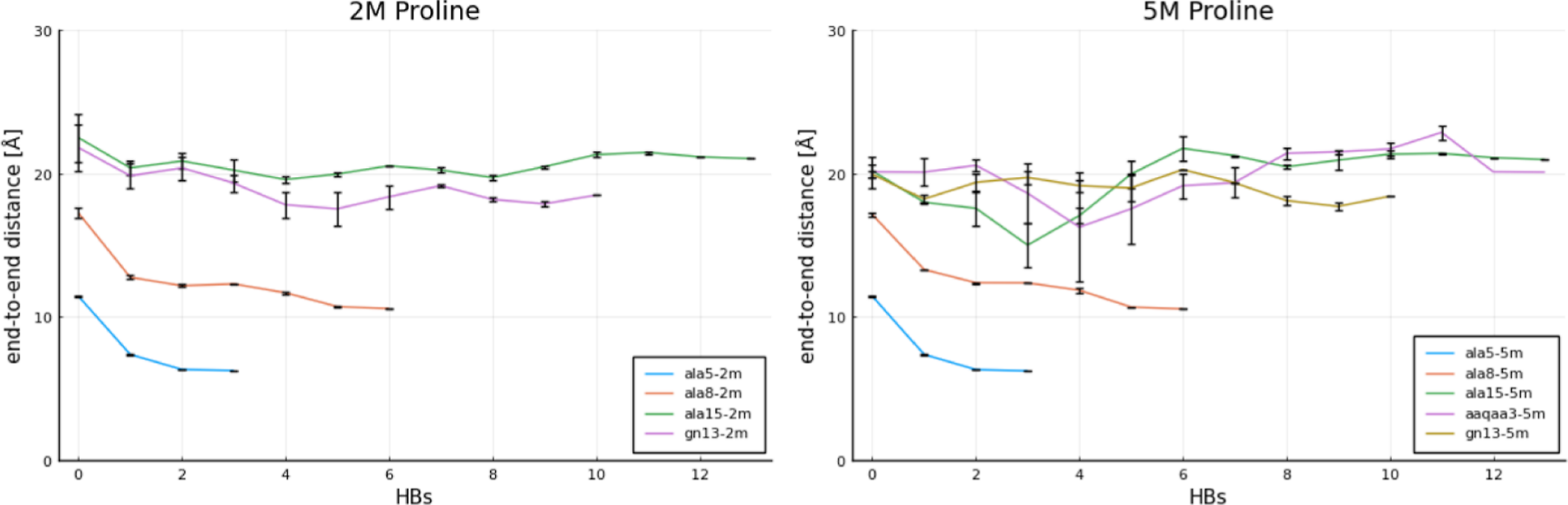
Movement of the center of mass *x*_*i*_ of the end-to-end distance histogram with the changing number of HBs. Left: 2 M proline. Right: 5 M proline.

In the next step, the so-called global friction values were obtained using eq 8. Therein, the value of Δ*x* has been calculated like in ref (28) i.e., first by calculating the center of mass, *x*_*i*_ for each end-to-end distance histogram obtained for structures with a specific number of HBs and then via summation of squared values of Δ*x*_*i*_ obtained from crossing from *i* to *i* + 1 HBs. The evolution of *x*_*i*_ values is plotted in Figures 7 and 8 for each proline concentration, i.e., solvent viscosity.

By observing the plots in Figures 7 and 8, one can notice that the values of *x*_*i*_ do not change with increasing proline concentration only in the case of smaller peptides. Similar conclusions can be reached by comparing the heat maps in Figure 2, where almost the same folding pathways appear to be present at 0 and 5 M proline. However, in the case of longer peptides, the values of Δ*x*_*i*_ increase significantly with the proline concentration. This shows a clear impact of proline on their folding pathways, which was also reflected in different heat maps obtained but also in the lack of equilibration between simulations for the same peptide originating from its “e” and “h” configurations; see Figure 3. Therein, some unfolded configurations have not been visited by a protein starting from its perfectly helical state (“h”) and some folded configurations have not been visited by a protein starting from its extended state (“e”).

Finally, we discuss the effect of concurrent folding pathways on the values of *x*_*i*_ and Δ*x*_*i*_. Concurrently occurring folding pathways have been illustrated for ALA8 in Figure 2; see arrows and lines for simulations at 5 M proline starting from the “e” state. This gets more complex with increasing length of the peptide, as can be seen in additional figures in the Supporting Information, see Figure S24 therein. The complex energy landscape at intermediate numbers of HBs suggests that there are many potential folding pathways which take quite diverse trajectories through the end-to-end distance/HB space. Nevertheless, in the case of homological peptide series, like the one studied here, additional folding pathways in the case of larger peptides add up to the already existing ones for their smaller counterparts. This has an effect on overall Δ*x*, which is analogous to summing up additional electrical resistances connected in parallel. This is because the value of Δ*x* is calculated through a weighted average (center of mass approach). Therefore, each occurrence of an additional compounding folding pathway is expected to manifest in slightly larger values of *x*_*i*_ and Δ*x* and, consequently, to decrease global friction coefficients. This effect is particularly visible for the ALA15 peptide in Figure 8 at 5 M proline. Interestingly, however, the overall changes in Δ*x* are not only balanced but become offset by particularly small unfolding rates of ALA15 at 5 M. This consequently leads to the largest values of global internal friction coefficients for this peptide.

**Figure 9:**
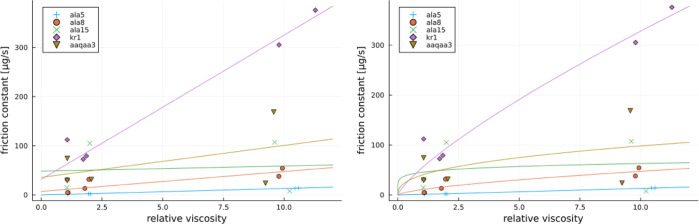
Global friction estimate as a function of relative viscosity for studied peptides. On the left with a linear fit and on the right with a power law fit.

**Figure 10:**
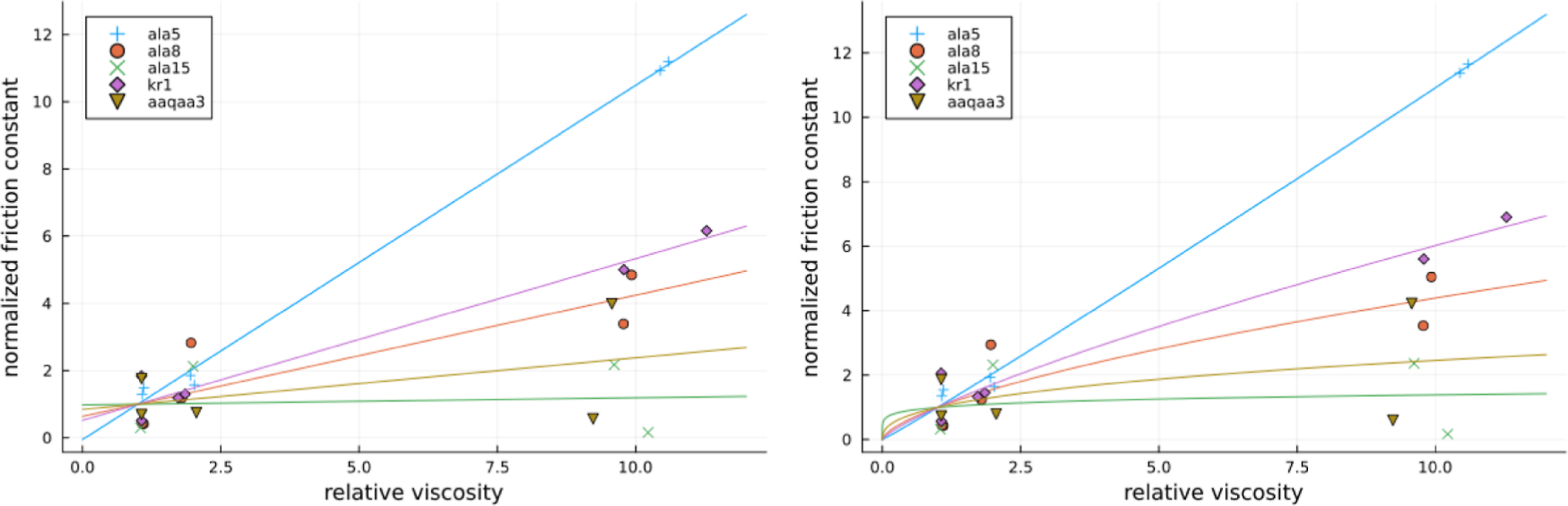
Normalized global friction estimate as a function of relative viscosity for studied peptides. On the left with a linear fit and on the right a power law fit.

The resulting values of Δ*x* and global friction coefficients for all peptides are tabulated in Table 4. We would like to notice that the results for no proline are very similar to the results previously published by Wosztyl et al.^28^ since the same set of data was reanalyzed but by a different operator. This is important since it establishes credibility of the current analysis. Global friction coefficients at various relative viscosities are plotted in Figures 9 and 10. Therein, the issues arising and discussed in the case of plots of the *x*_*i*_ values manifest themselves as follows. Notably, larger scatter between the points at higher viscosities is a consequence of a lack of equilibration for the simulations conducted under such conditions. From this perspective, there is also no improvements obtained for power law vs linear fits. Only sufficiently longer simulations would address whether there is a clear need for power law fits. Consequently, we use linear fits to obtain the global internal friction at zero viscosity. Table 5 shows the results, which vary between 0 and 50 μg/s, and will be discussed below together with the results of local internal friction. Noteworthy, for the fits in the case of ALA15 and AAQ peptides at 2 M proline, only a smaller value of friction coefficient was used. This was due to a large difference between upper and lower limits of the friction coefficients obtained for the “e” and “h” trajectories, respectively, in these cases.

**Table 4:** Values of Global Friction Coefficients and, Respective, Δ*x* Used for Their Calculations

peptide	proline	global friction coeff. [μg/s]	Δ*x* [Å]
Ala5	5 M	14.0 ± 0.2	4.22 ± 0.01
Ala8		46.2 ± 8.2	4.17 ± 0.16
Ala15		57.6 ± 49.7	6.24 ± 2.09
AAQAA3		96.4 ± 72.4	5.31 ± 2.43
KR1		340 ± 35	3.45 ± 0.24
Ala5	2 M	2.17 ± 0.18	4.20 ± 0.04
Ala8		22.5 ± 9.2	4.68 ± 0.25
Ala15		185 ± 80	2.98 ± 1.03
AAQAA3		317 ± 285	2.34 ± 1.47
KR1		76.0 ± 3.3	3.49 ± 0.12
Ala5	0 M	1.76 ± 0.12	4.31 ± 0.01
Ala8		4.72 ± 0.07	4.34 ± 0.15
Ala15		23.3 ± 8.4	2.73 ± 0.29
AAQAA3		51.9 ± 22.5	2.53 ± 0.62
KR1		71.7 ± 40.5	2.47 ± 0.64
Ala21		47.0 ± 9.2	3.65 ± 0.08

**Table 5:** Global Internal Friction for All Studied Cases, Calculated as the *y*-Axis Intercept at Vanishing Viscosity

peptide	global internal friction [μg/s]
Ala5	0.100 ± 0.100
Ala8	7.18 ± 6.69
Ala15	46.9 ± 35.1
KR1	31.7 ± 18.4
(AAQAA)_3_	38.3 ± 38.3

### Comparison between Local and Global Internal Friction

The obtained values of local internal friction were between 4 and 28 ng/s with the smallest value for the ALA5 peptide and the largest value for the ALA15 peptide; see Table 3. Furthermore, the rate of growth of the local friction coefficient with viscosity, see Figure 6, depends on the structure. It is the fastest for the ALA15 peptide, similar for ALA5 and ALA8 peptides, and much smaller for the AAQ and KR1 peptides, which prefer to stay within unfolded conformations. Taken together, this means that comprehensive measurements of friction coefficients at various solutions are needed to understand and correlate this parameter with structural properties of each given peptide.

The obtained values of global internal friction were between 0.1 and 46.9 μg/s with the smallest value for the ALA5 peptide and the largest value for the ALA15 peptide; see Table 5. Such global internal friction even if divided by a total number of HBs within a given protein yields values being 1 or 2 orders of magnitude larger than those in the case of local internal friction. Nevertheless, the order of peptides obtained in the case of local and global internal friction is similar. The ALA5 and ALA8 peptides show the smallest internal friction. The KR1 and AAQ peptides are in between. The ALA15 peptide displays the highest internal friction. Thus, two different methodologies qualitatively yield comparable results. The actual values, in micrograms per second, have yet to be experimentally measured.

Comparisons with existing experiments are possible only in terms of appropriate reconfiguration times. The dwell times associated with local internal friction, as obtained through eq 6, are between 10 and 70 ps. Correspondingly, supposing 2 orders of magnitude larger values for the global friction, we obtain the times between 0.1 and 10 ns. The times that would be associated with our values of global friction are indeed very similar as in the case of reconfiguration times for dihedral angle isomerization, τ_dai_. For example, in the case of a minimal butane-like molecule, the value of τ_dai_ of 10 ns was obtained in the literature.^41^ In the case of 66 residue-long *Thermotoga maritima*, Cold Shock Protein (PDB access code 1G6P) τ_dai_ was ca. 30 ns.^26^ In both cases, no dependence of τ_dai_ on viscosity was observed. On the other hand, we have also calculated the shortest reconfiguration times associated with a single, ensemble-averaged HB formation/rupture. We obtained values ranging from 0.2 ns for ALA5 to 2.4 ns for ALA15 and 10 ns for ALA21. Indeed, dihedral angle isomerizations can be largely responsible for global internal friction and these values correlate as well with ensemble-averaged HB reconfigurations. In contrary, the values of local internal friction associated with dwell times of single, ensemble-averaged HB, are much smaller and might not even be visible within the experiments if dihedral angle isomerization would setup the shortest accessible time scale.

## Conclusions

Guided by potential applications for AFM measurements, we have presented two approaches to model the processes relevant for obtaining friction coefficients associated with folding dynamics of several examples of α-helical peptides in the presence of proline, i.e., one of the typical osmolytes.

First, we concentrated on the dwell times of an ensemble-averaged HB as the source of energy dissipation, leading to *local friction coefficients*. Using the dwell time analysis method, previously applied in the force-clamp SMFS with AFM, we obtained a relevant time scale associated with lasting of a single HB averaged over all HBs within each given peptide. These values were later used to calculate local internal friction coefficients at various viscosities determined by proline concentrations.

Second, in order to avoid direct applications of the Stokes law (used earlier for *local friction coefficients*), a different approach was proposed to calculate friction coefficients arising from larger molecular conformational changes. This approach was based on thermodynamic parameters, such as Δ*x*, which are potentially observable in the FC-AFM experiments. The friction coefficients, which related to folding/unfolding conformational transitions, were named *global friction coefficients*. From the thermodynamic analysis, we concluded that evolution of global friction coefficients with viscosity was weakly dependent on the number of concurrently occurring folding pathways, i.e., evolution of the Δ*x* values. It was rather dominated by a thermodynamically calculated stabilizing effect of proline on the folded states occurring particularly at large viscosities, i.e., reaction rate constants.

By fitting the data with a linear dependence of friction coefficients with viscosity, we obtained local and global internal friction, i.e., friction coefficients at zero viscosities, respectively. The time scales associated with local internal friction were tens of picoseconds and relevant internal friction varied between 4 and 28 ng/s. Global internal friction values were between 0.1 and 46.9 μg/s, i.e., 2–3 orders of magnitude larger than local internal friction. Therefore, rescaling global internal friction by a given number of HBs for each peptide did not bring these values into the proximity of local friction. However, local and global internal frictions showed the same trends and a clear dependency on the structure of the studied peptide.

Concerning the role of proline, it has become evident from thermodynamics (reaction rates) as well as stability conditions (simplified folding funnels) that proline stabilizes the folded state. Furthermore, in the case of peptides with more than eight residues, proline also stabilized partially folded states. Such stabilization was nonspecific, i.e., without any preference for particular residues. Furthermore, proline slowed the folding dynamics by introducing larger viscosity and thus thermodynamic hindrance in exploring a full energy landscape. Consequently, the peptides started from extended conformations explored less of the folded states and vice versa. Finally, the addition of proline increased populations of all individual helical HBs. This effect goes beyond that previously discussed and is likely due to changing solute–solvent interactions.

Comparison with literature pointed out that our values of global internal friction (in kg/s) correlate with internal friction (in seconds) attributed to dihedral angle isomerizations in short molecules containing only several dihedral bonds. They also correlated with ensemble-averaged reconfiguration times obtained from autocorrelation of the number of HBs with time throughout the simulations. In contrast, the values of local internal friction (in kg/s), when expressed into appropriate times, were 2 orders of magnitude smaller than all of the aforementioned times. Thus, such small values of local internal friction might not even be visible within the future AFM experiments if dihedral angle isomerizations setup the shortest accessible time scale.

## Supporting Information Available

Initial folding analysis (RMSDH plots and heat maps) for all studied peptides; detailed description and derivation of local friction estimates; itemization of issues with usage of RMSDH plots for local friction calculations; description for obtaining dwell times for local friction calculations; and additional descriptions of vital parameters for global friction calculations. (PDF)
