# Supplementary material for: Effects of Proline on Internal Friction in Simulated Folding Dynamics of Several Alanine-Based α-Helical Peptides
Source: J Phys Chem B. 2024 Apr 12;128(16):3856–69. doi: 10.1021/acs.jpcb.4c00623 (PMC11056985; doi:10.1021/acs.jpcb.4c00623)
Supplement: Supplementary file 1 — jp4c00623_si_001.pdf [file jp4c00623_si_001.pdf]

Supporting Information for Publication: The Effects of Proline on Internal Friction in Simulated Folding Dynamics of Several Alanine-based α-helical Peptides.

Adam Świątek,[†] Krzysztof Kuczera,[*,‡,¶] and Robert Szoszkiewicz[*,†]

†*Faculty of Chemistry, Biological and Chemical Research Centre, University of Warsaw, Żwirki i Wigury 101, 02-089 Warsaw, Poland.*

‡*Department of Chemistry, The University of Kansas, Lawrence, Kansas 66045, USA.*

¶*Department of Molecular Biosciences, The University of Kansas, Lawrence, Kansas 66045, USA.*

E-mail: kkuczera@ku.edu; rszoszkiewicz@chem.uw.edu.pl

Initial folding analysis

Some additional data related to visualization and initial analysis of the MD trajectories for the studied peptides are presented below. We start by plotting RMSDH variations with time, see Figs.S1 - S5.

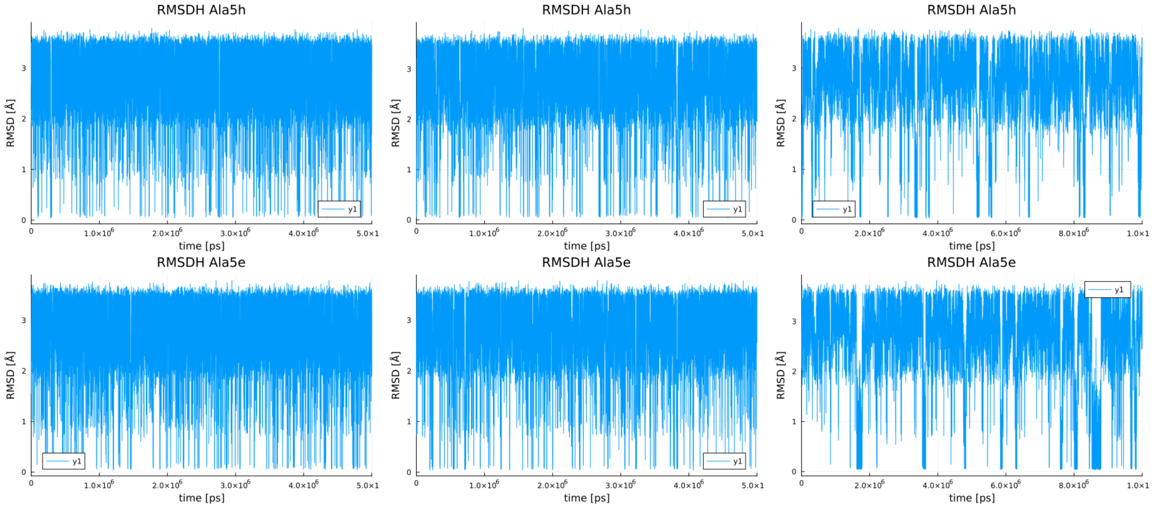

Figure S1: RMSDH timeseries for ALA5. The top row is "h" configurations, the bottom row "e". The left column is in pure water, the middle in 2M proline and the right in 5M proline.

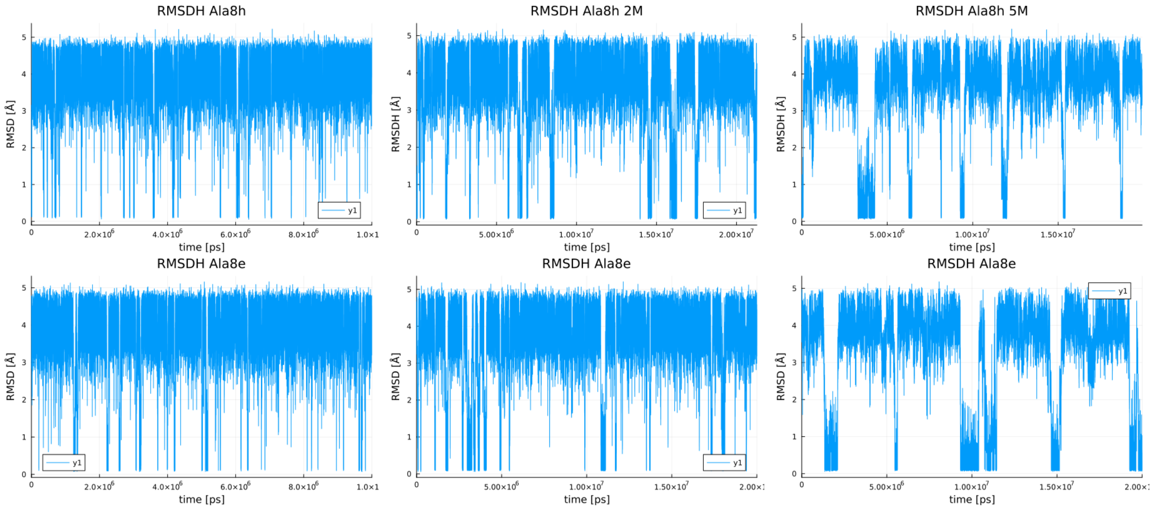

Figure S2: RMSDH timeseries for ALA8. The top row is "h" configurations, the bottom row "e". The left column is in pure water, the middle in 2M proline and the right in 5M proline.

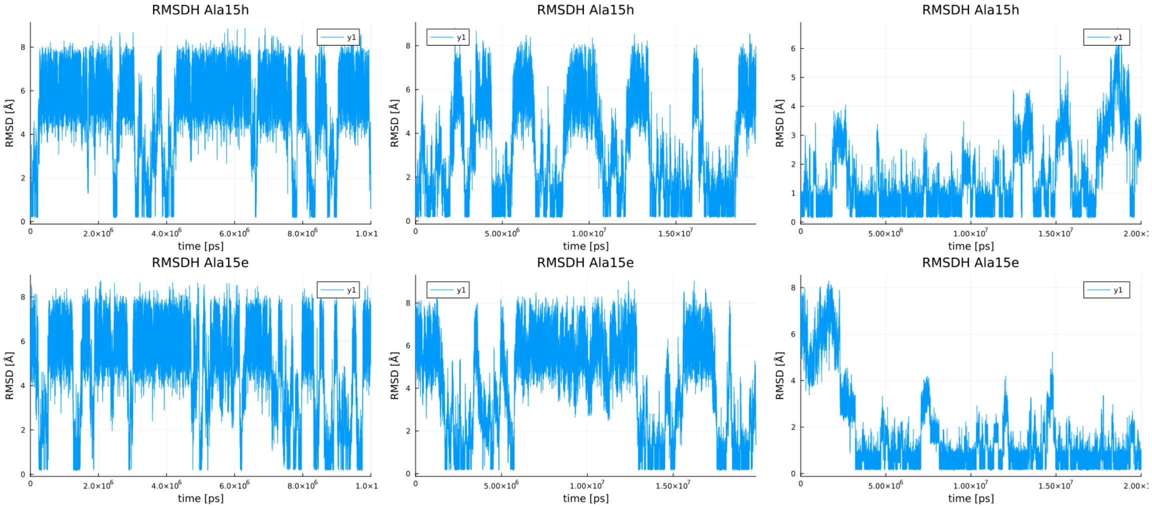

Figure S3: RMSDH timeseries for ALA15. The top row is "h" configurations, the bottom row "e". The left column is in pure water, the middle in 2M proline and the right in 5M proline.

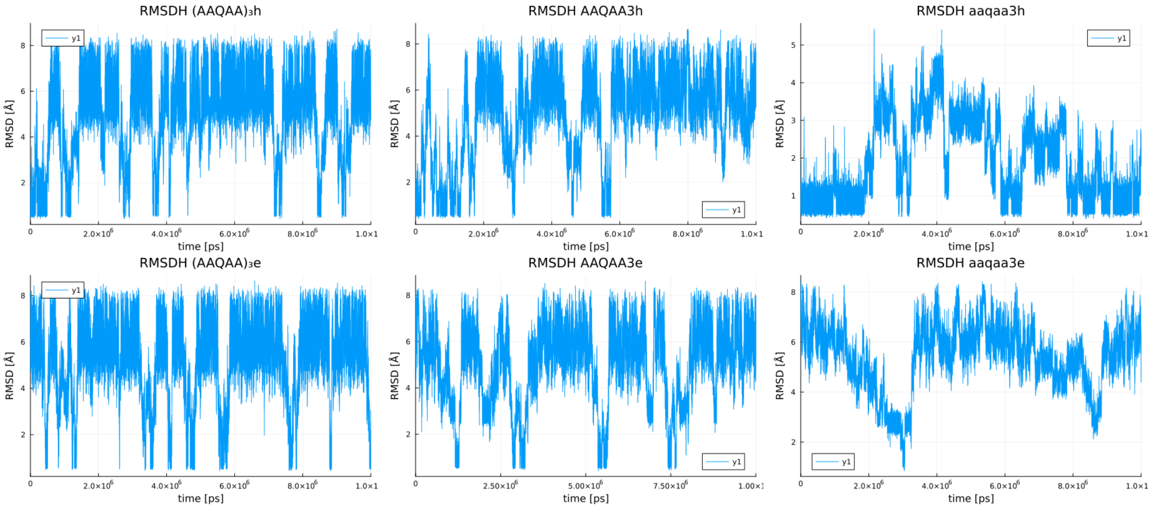

Figure S4: RMSDH timeseries for $(AAQAA)_3$. The top row is "h" configurations, the bottom row "e". The left column is in pure water, the middle in 2M proline and the right in 5M proline.

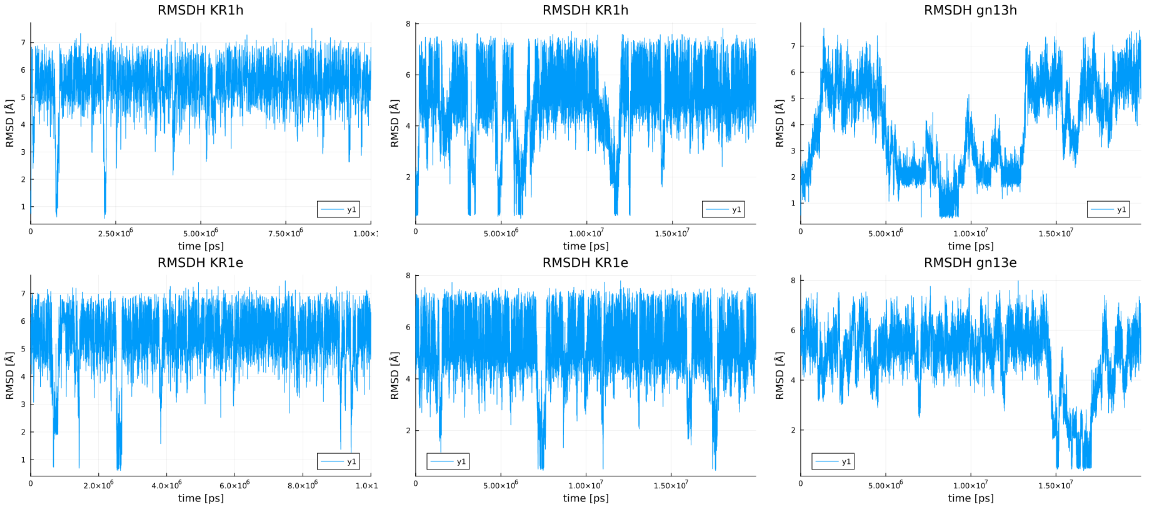

Figure S5: RMSDH timeseries for KR1. The top row is "h" configurations, the bottom row "e". The left column is in pure water, the middle in 2M proline and the right in 5M proline.

Next, we plot the free energy surfaces as function of HB number and end-to-end distance, see Figs. S6 - S10.

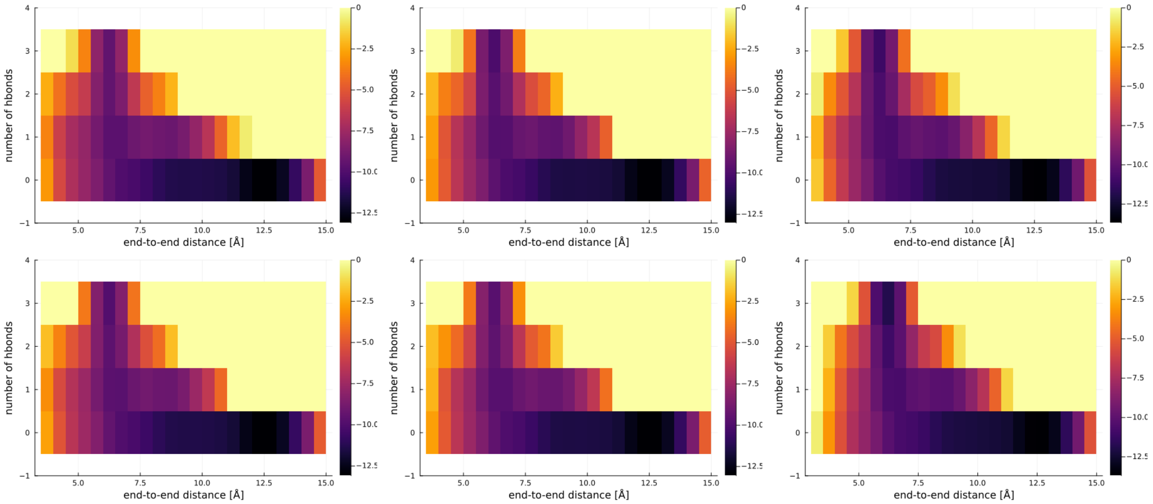

Figure S6: Representation of the free energy surface for ALA5. The top row is "h" configurations, the bottom row "e". The left column is in pure water, the middle in 2M proline and the right in 5M proline.

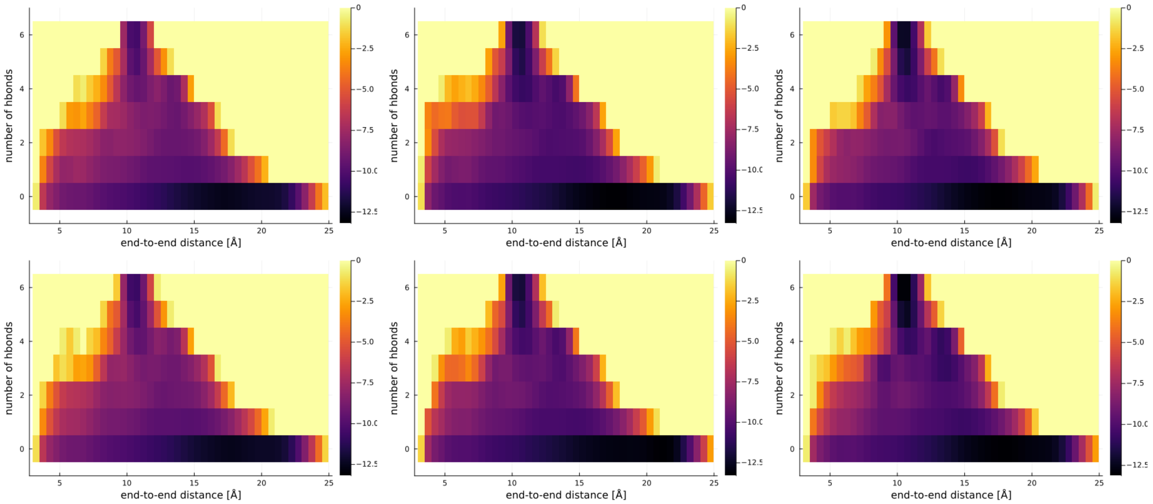

Figure S7: Representation of the free energy surface for ALA8. The top row is "h" configurations, the bottom row "e". The left column is in pure water, the middle in 2M proline and the right in 5M proline.

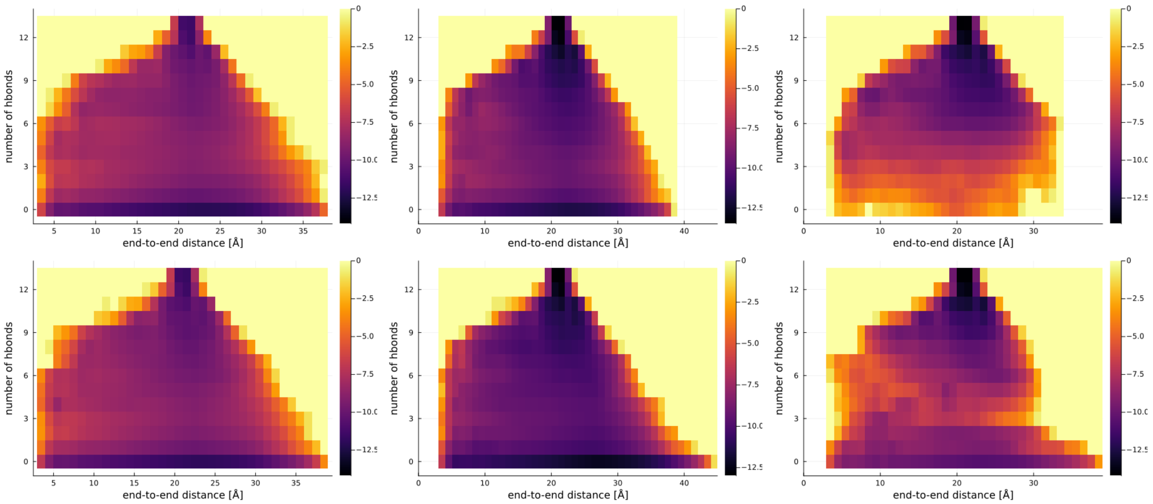

Figure S8: Representation of the free energy surface for ALA15. The top row is "h" configurations, the bottom row "e". The left column is in pure water, the middle in 2M proline and the right in 5M proline.

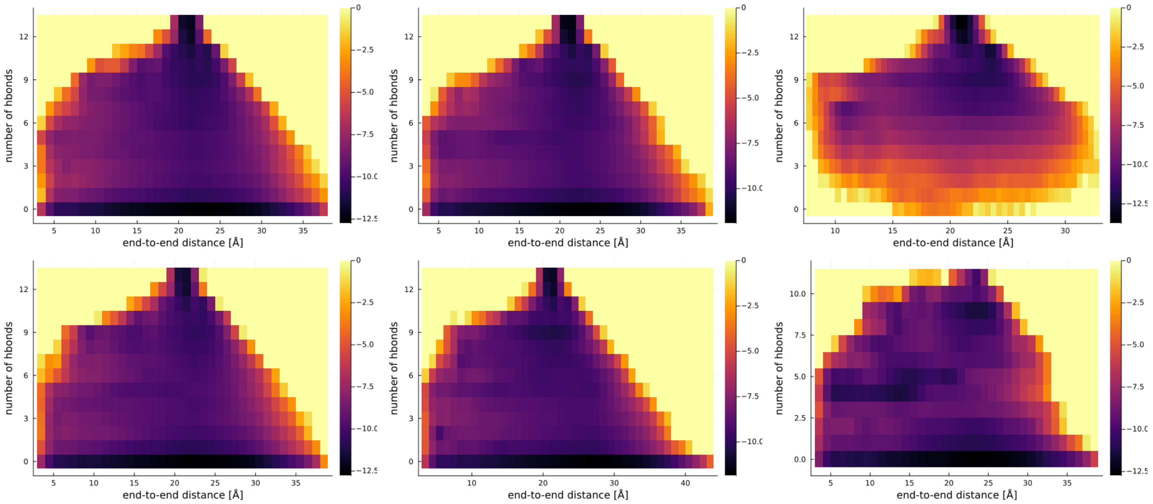

Figure S9: Representation of the free energy surface for $(AAQAA)_3$. The top row is "h" configurations, the bottom row "e". The left column is in pure water, the middle in 2M proline and the right in 5M proline.

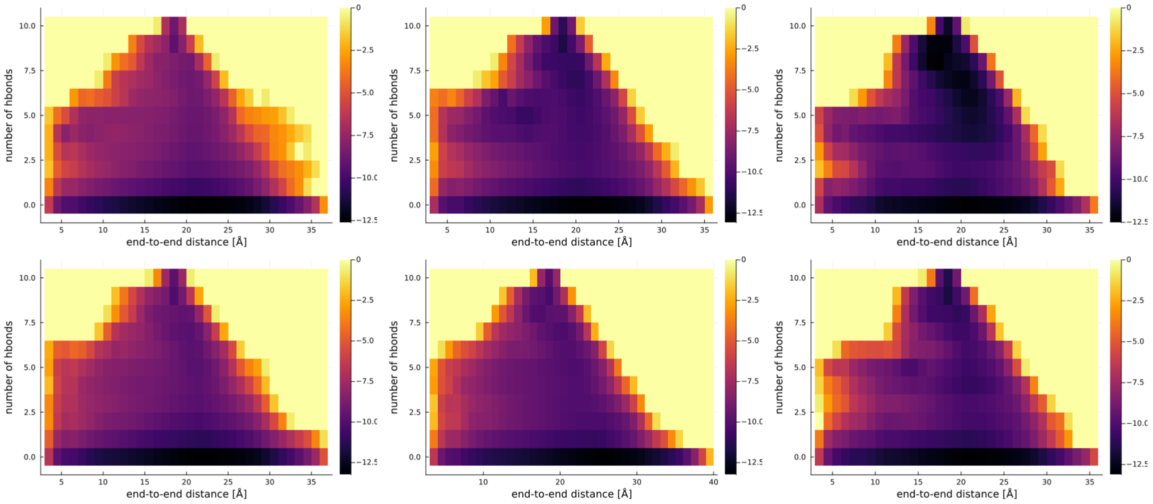

Figure S10: Representation of the free energy surface for KR1. The top row is "h" configurations, the bottom row "e". The left column is in pure water, the middle in 2M proline and the right in 5M proline.

Local friction estimates

Helix distance change, δ

The variable δ is the increase or decrease in the helix length with the creation or destruction of a single hydrogen bond (HB). To estimate its value we considered histograms showing the distance between an O-N pair in residues which may form a helical hydrogen bond while requiring that a neighboring pair (along the helix) is currently bonded. Figs. S11, S12 show two typical examples of such histograms obtained for Ala8h in pure water in the so-called "forward" and "reverse" cases, see captions of the figures. These histograms are bimodal. The first maximum corresponds to a HB between considered residues. We call this maximum d_{HB}. The second maximum corresponds to the most likely distance, when no HB is present. Due to an unknown nature of this distribution, we calculate its weighted average and call it d. When comparing Fig. S11 and Fig. S12 one can notice that the position of the first maximum does not change much. However, the position of the second peak is quite affected. The shift of the second peak toward larger values between HB3 and HB2 in Figs. S11 & S12 can be explained by the fact that HB2 is closer to the peptide terminus and thus has somewhat higher conformational flexibility than HB3. Helix length change in folding dynamics of alanine polypeptides has also been showed in Ref.[1] to depend on whether the helix extends towards the N-terminal or the C-terminal. Therefore, we average the corresponding values of d for the two directions, to yield d_{ave}. Next, we calculate δ as:

$$\delta = d_{ave} - d_{HB}$$

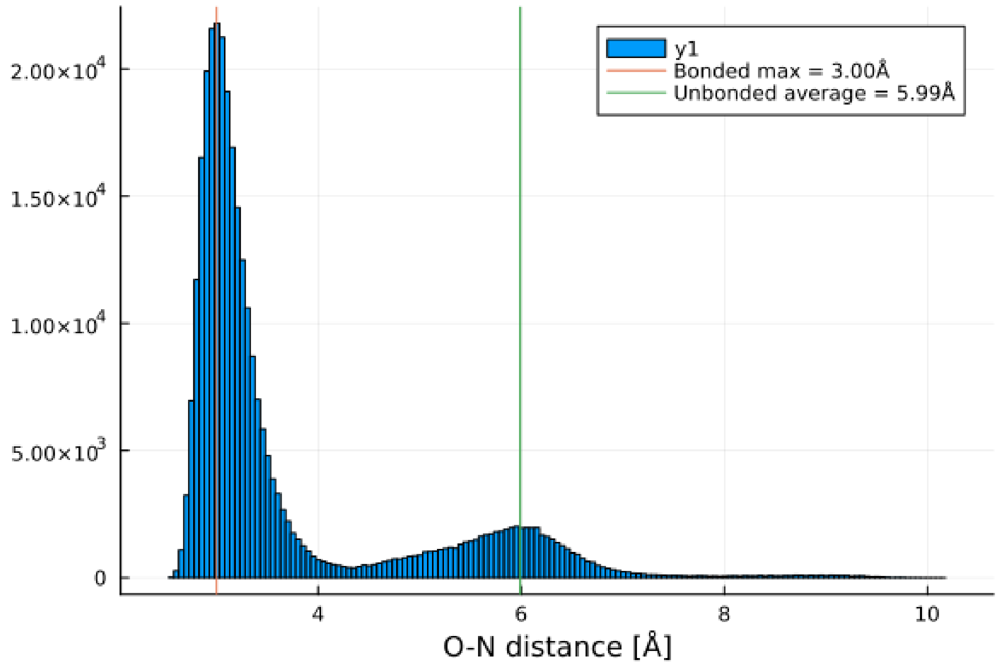

Figure S11: Distribution of distances in Ala8h0m of the third hydrogen bond along the helix considering that the second HB already exists. The HBs are counted from the N to C termini of the peptide.

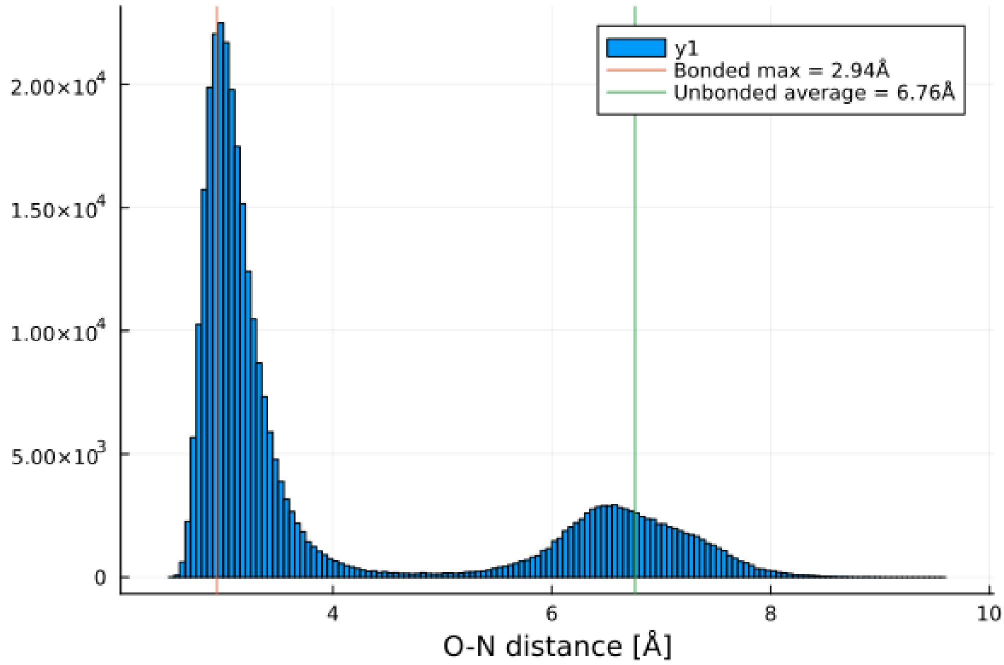

Figure S12: Distribution of distances in Ala8h0m of the second hydrogen bond along the helix considering that the third HB already exists. This is the reverse of Fig.S11.

All other histograms of this type, not shown here, show a remarkable similarity among all of our considered peptides and a low sensitivity to proline concentration. Such results for a significant fraction of studied cases are presented in Table S1. Errors, estimated at roughly 10 % arise from the averaging done to obtain the values of d_{ave}.

Table S1: Values of δ as used for estimating local friction for selected scenarios.

Peptide	Proline	δ [Å]
Ala8	5M	3.46
Ala8	2M	3.41
Ala5	0M	3.39
Ala8		3.43 ± 0.39
Ala15		3.38
Ala21		3.32
KR1		3.48
$(AAQAA)_3$		3.49

Issues in biexponential fits to RMSDH autocorrelation functions

Before settling on dwell times analysis as the method to obtain the shorter "local" relaxation times we attempted to extract these times from RMSD autocorrelation functions, because this is one of the most used proxies.

We are of the opinion that consistent failures of the RMSD approach originated from very small effect of local hydrogen bond formation on the overall RMSD for the protein, due to very small displacements of only several atoms in this process.

To start with the RMSD approach, we fitted a biexponential decay with a constant term[1] to the RMSD ACF using the Levenberg-Marquardt non-linear fitting algorithm. This typically gave results as in Fig. S13 with the fit term containing the shorter time carrying a very low weight to the extent that sometimes it failed to be resolved. In other words, the fit would converge to an amplitude of zero for one of the decay time constant.

Next approach consisted in taking the natural logarithm of the RMSD ACF, ln(ACF).

[1]The autocorrelation has mean 0 by definition, therefore an "exponential decay" in an autocorrelation won't actually tend to 0 but instead to slightly less, to offset the positive values at low times

At sufficiently long times, the ln(ACF) will asymptotically approach an affine function with slope dependent on the slowest decay time constant. That decay function can then be subtracted from the original ACF and the process iterated until all decay time constants are extracted. This approach however ran into the following issues. First, oscillations in the ACF caused it to dip below zero at moderate times and this made the ln(ACF) function diverge, see Furthermore, these oscillations couldn't be fitted with sine functions making it impossible to extract the slowest decay constant from its asymptotic behavior. Instead, a straight run in ln(ACF) had be chosen by hand just before the oscillation dominated zone. In a majority of cases a linear portion of the ln(ACF) function was clearly identifiable. Two representative fits of the slowest decay are shown in Figs. S14 and S15. Nevertheless, even a small change in the zone of linear fit caused large differences in subsequent fits. This indicated high sensitivity of the short times to any error in the linear fit of the longer times. In addition, the iterative method regularly revealed more than two exponential decays in the RMSD ACF. In other words, ln(ACF) failed to become linear after removing one decay. The third and further times also suffered even more from the errors made on earlier iterations. These issues lead to questions about which of the short times should be used.

Ultimately, extraction of low-amplitude features using increasingly more refined techniques produced substantial errors. Therefore, we started to search for a more appropriate proxy for the single-hydrogen-bond-scale dynamics. To this we opted to use dwell time analysis.

Dwell times of single hydrogen bonds

Dwell time analysis to learn about the rate (and its inverse τ) for a single hydrogen bond formation/rupture has been applied here. Dwell times have been calculated as the lifetimes of a particular non-terminal HB from within each given peptide throughout a whole MD trajectory. For example, for ALA8 peptide the 4th HB was taken, while for ALA15, the 6th HB was taken. All such choices are listed in Tab. S3.

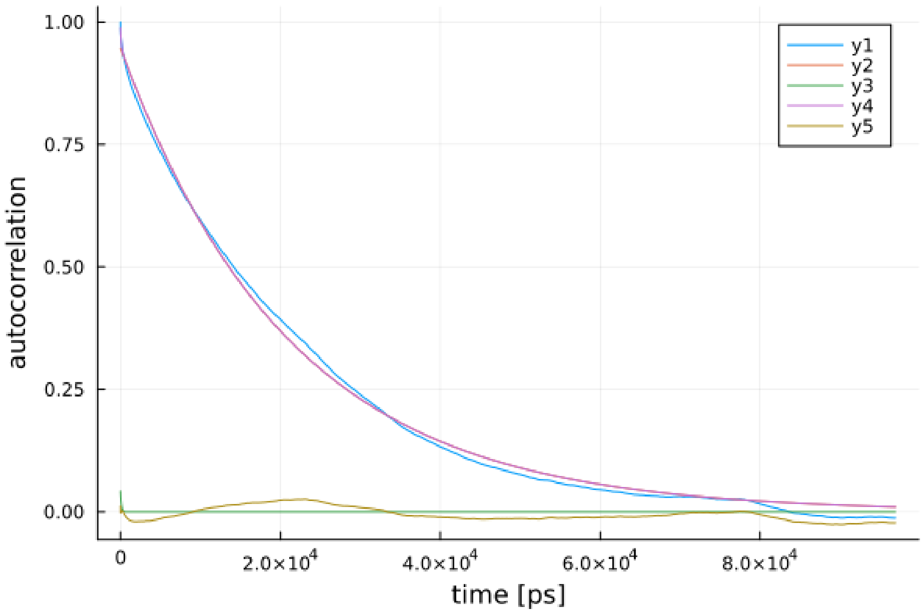

Figure S13: Biexponential decay fit to Ala5h in 5M proline ACF. The yellow trace is the residue. This example shows the very small amplitude of quicker decay characteristic of such fits.

To extract an appropriate value of τ logarithmic dwell time histograms are constructed by binning the logarithms of dwell times and taking the square root of the counts. This approach has been described already in Ref.[2] and its application to single disulfide bonds can be found in Ref.[3]. Using the square root of the counts causes count errors in each bin to be constant.[2] Most importantly, however, binning logarithms instead of the times allows for visual separation of the decay time constants. This is because exponentially-distributed decays have maxima at zero time with visually inseparable decays constants, while logarithmic histograms show separated maxima at positions defined by the decay constants. An example of such histograms is shown in Fig. S16(LEFT). Therein, a bin size of 0.23 in the scale of natural logarithm of time and with time in ps. It was taken after comparing several bin sizes to distribute the data sufficiently well.[3] Later, the same bin size was applied to all of our data.

In all of our histograms, the raw dwell time data had quantization error arising from MD simulation timestep of 2 ps. Examining the data in Fig. S16(LEFT) one can see

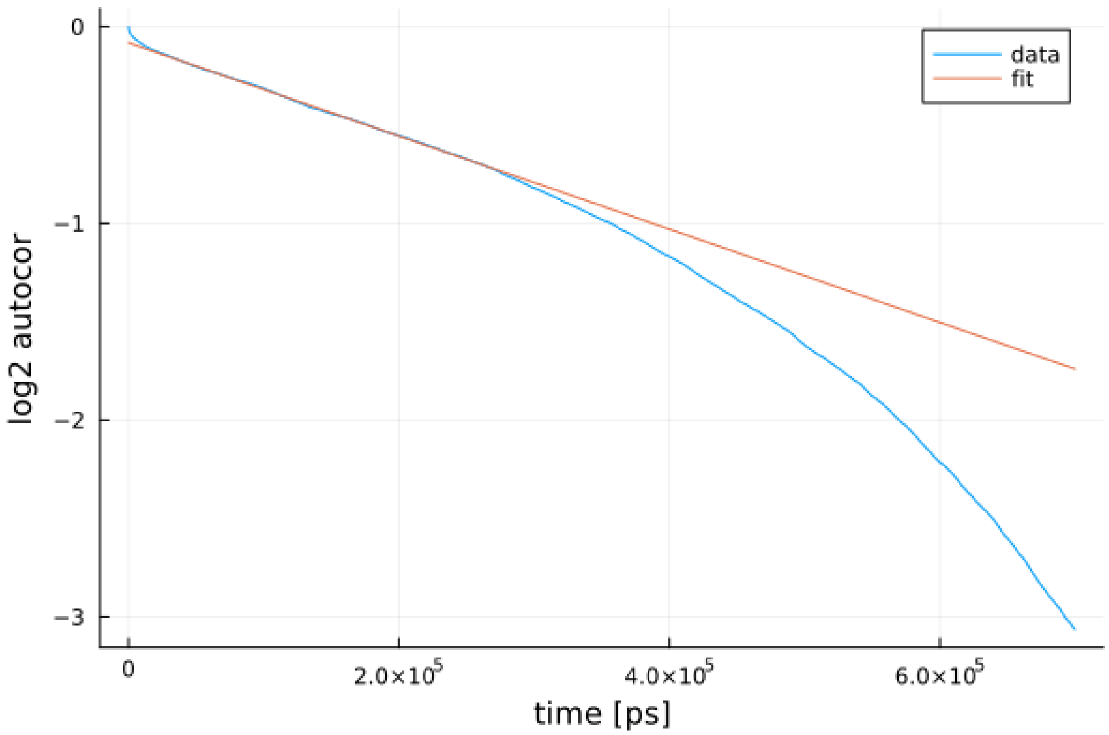

Figure S14: Ala15h with 2M proline

substantial fluctuations of the counts within the bins extending to about at least "3" in the lateral units. This corresponds to the time of about 20 ps. Therefore, to better visualize the distribution some noise has been applied to the dwell times by subtracting a random constantly-distributed number in the range (0, 2 ps) from each dwell time. This corresponds to an idea that each dwell time detected within 2 ps time steps could in reality exist for shorter times than that. The noise-corrected data are presented in Fig. S16(RIGHT). One can see that noise addition does not change the maximum of the histogram, but seems to deal well with the quantization error. Noteworthy, this approach approximates the data at shorter times according to a predicted Poissonian-like distribution for one event occurring within each given dwell time. Consequently, the same procedure has been applied to all of our data.

Two other examples of the noise-corrected logarithmic dwell time histograms for other peptides are presented and described in Figs. S17 and S18. To fit the decay time constants, and consequently extract the times τ mono- and bi-exponential fits have been applied to

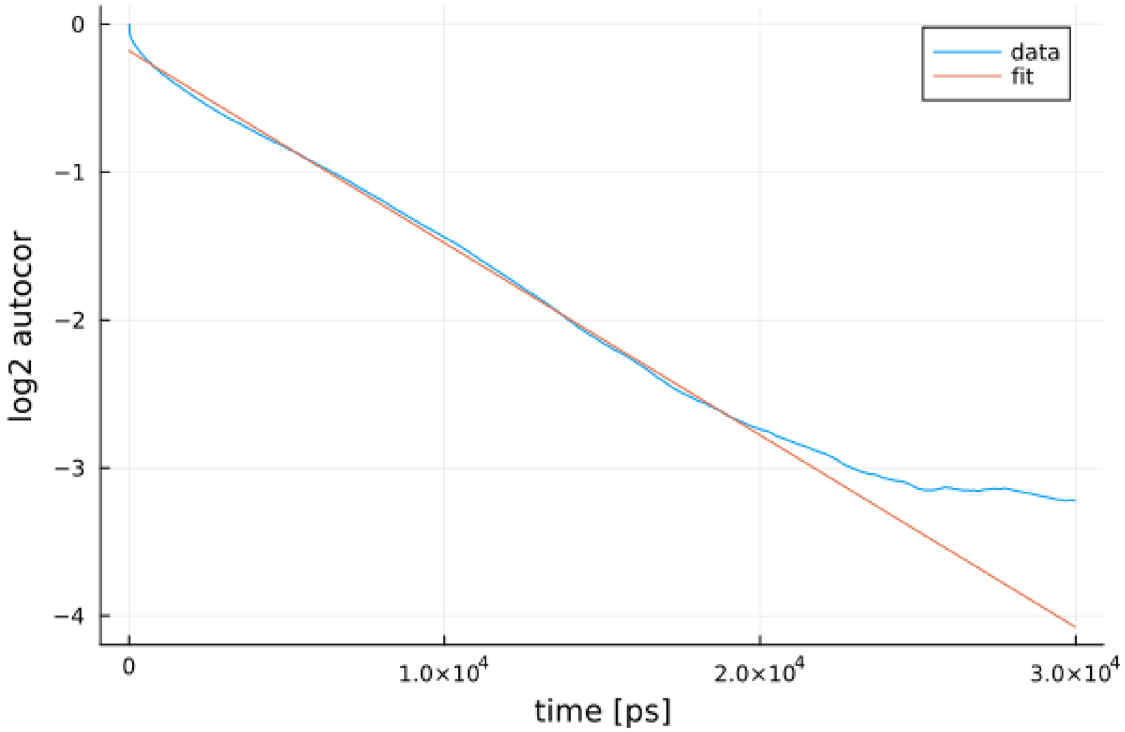

Figure S15: Ala8h in water

such histograms. Comparing the presented mono- and bi-exponential fits in Figs. S17 - S19 one concludes that a mono-exponential fitting is not sufficient, even in the case of letting the overall number of events N vary. Therefore, we use bi-exponential fits, which produced much better results. The data are presented in the Table S2 below.

In the Table S2 each of the dwell times is an average obtained for "extended" and "helical" simulations. The errors are maximum errors, i.e., half of the difference between the data. While on the first glance we do not find physical reason behind a bi-exponential fitting, the shorter of the dwell times are always in the range of our simulation step. Therefore, they relate to the events, which have not been detected properly and as such cannot be further analysed. Consequently, we will use a bi-exponential fitting and the longer of the obtained dwell times as the times τ.

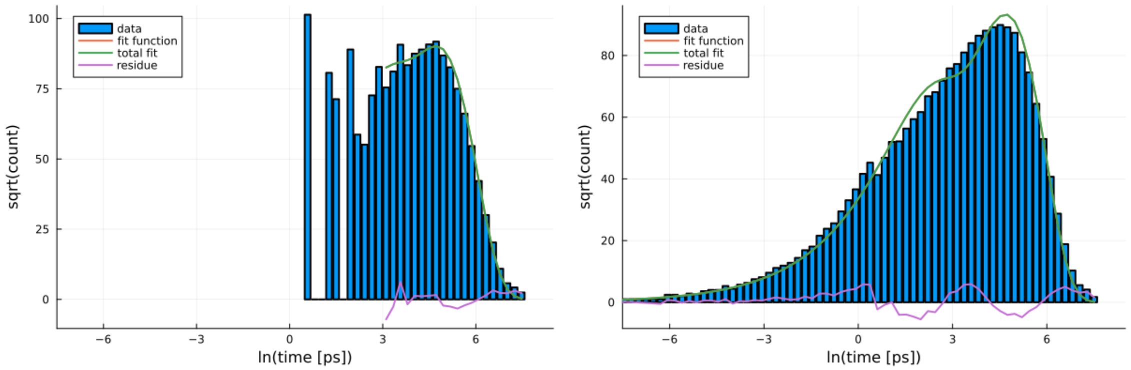

Figure S16: Example fit of a histogram of presumed exponential-distributed dwell times with logarithmic x-axis and the square root of the counts. The represented case is the 8th HB in Ala21h in pure water. On the LEFT the raw data with visible quantization error occurring on the time scale of several ps (corresponding roughly to "1" in the log(time) scale). A bi-exponential fit in the range $(3, \infty)$ is also added. On the RIGHT the same data with noise applied and a bi-exponential fit within the entire range.

Table S2: The shorter and longer dwell times from bi-exponential fits to logarithmic dwell time histograms, such as in Figs.S17 and S18.

Peptide	Proline	Dwell time (short) [ps]	Dwell time (long) [ps]
Ala5	5M	3.70 ± 0.12	36.63 ± 0.72
Ala8		5.06 ± 0.39	81.14 ± 6.3
Ala15		10.94 ± 0.07	309.43 ± 11.63
KR1		3.93 ± 0.05	73.19 ± 1.59
AAQAA3		2.73 ± 0.87	61.68 ± 21.20
Ala5	2M	3.29 ± 0.15	16.99 ± 0.04
Ala8		3.77 ± 0.10	41.5 ± 0.53
Ala15		6.33 ± 0.14	135.4 ± 0.05
KR1		3.39 ± 0.12	44.52 ± 4.61
AAQAA3		1.97	63.8 ± 2.6
Ala5	0M	2.80 ± 0.01	12.80 ± 0.03
Ala8		4.09 ± 0.28	28.26 ± 0.21
Ala15		6.47 ± 0.31	86.5 ± 1.54
KR1		7.21 ± 0.82	109.38 ± 1.59
AAQAA3		1.80 ± 0.03	27.54 ± 0.07

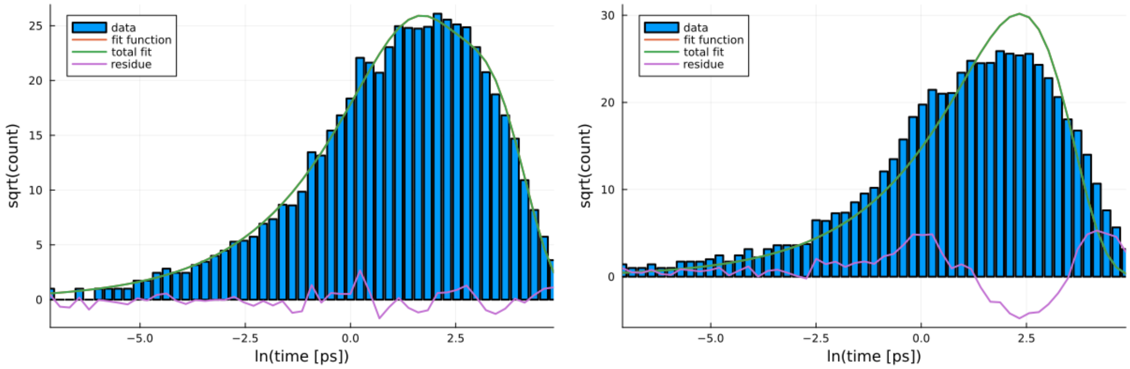

Figure S17: On the LEFT, an example fit of a histogram of presumed exponential-distributed dwell times with logarithmic x-axis. On the RIGHT the mono-exponential fit to the same data. The represented case is Ala5h in 2M proline

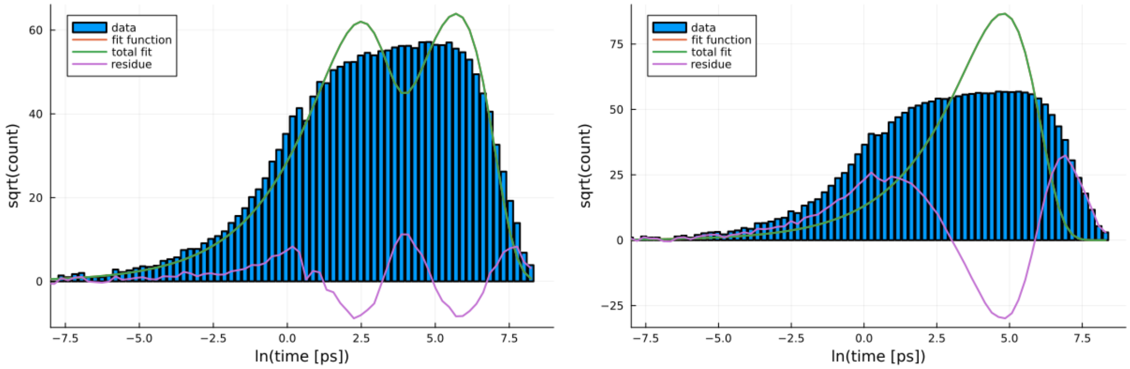

Figure S18: On the LEFT, an example fit of a histogram of presumed exponential-distributed dwell times with logarithmic x-axis. On the RIGHT the mono-exponential fit to the same data. The represented case is Ala15h in 5M proline. This case exemplifies a bad fit which can occur when the distribution has a flat top and consequently needs to be fitted with more than two exponentials.

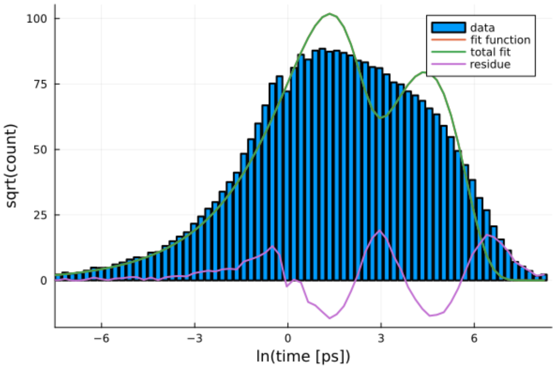

Figure S19: A poor double-exponential fit on AAQAA3 in 5M proline.

Table S3: Helical hydrogen bonds used for dwell time calculation

Peptide	Hbond
Ala5	1ALA-5ALA
Ala8	3ALA-7ALA
Ala15	5ALA-9ALA
Ala21	7ALA-11ALA
AAQAA3	5ALA-9ALA
KR1	5GLN-9ALA

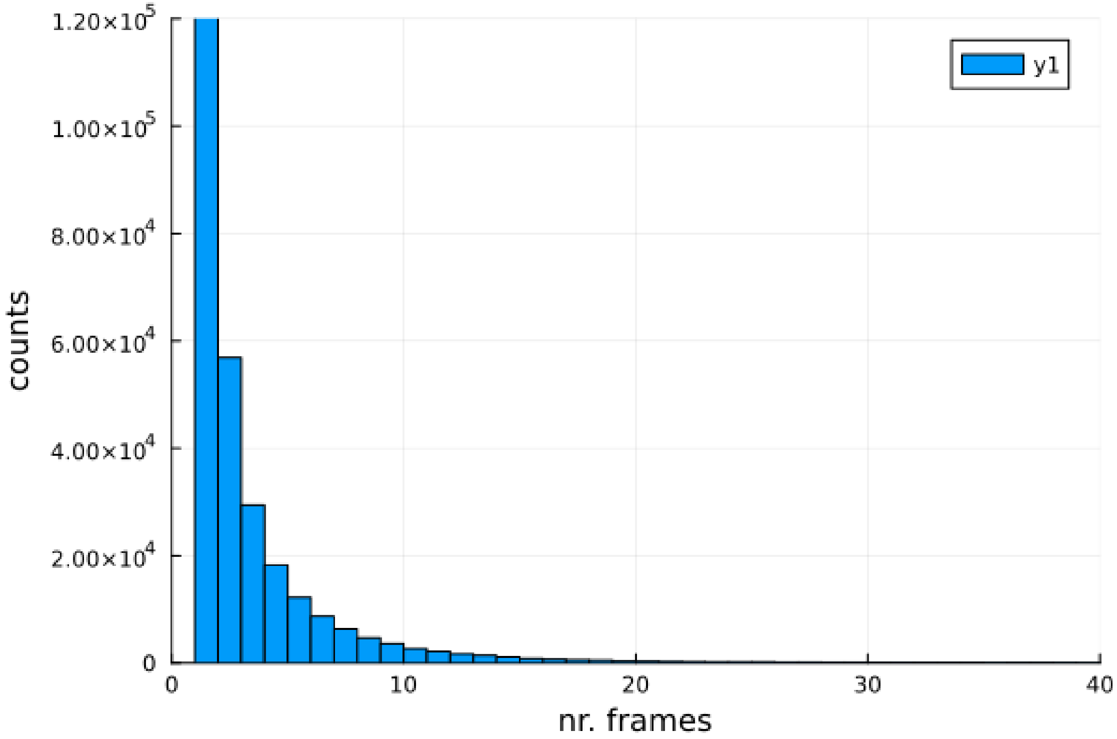

Figure S20: The beginning of the histogram showing the distribution of run lengths, in number of simulation frames. Here the smoothing algorithm was not applied. The depicted protein is Ala8e.

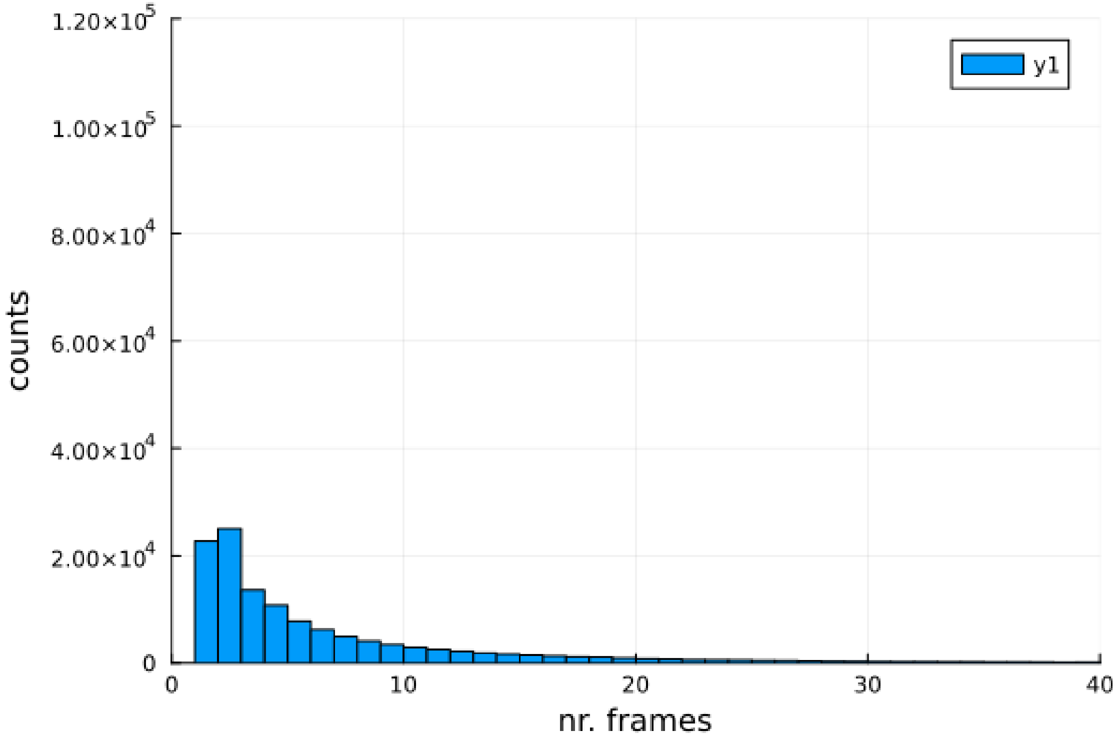

Figure S21: The beginning of the histogram showing the distribution of run lengths, in number of simulation frames. The data was smoothed by eliminating cases where the number of hydrogen bonds changed, and then reverted in the next frame. The depicted protein is Ala8e.

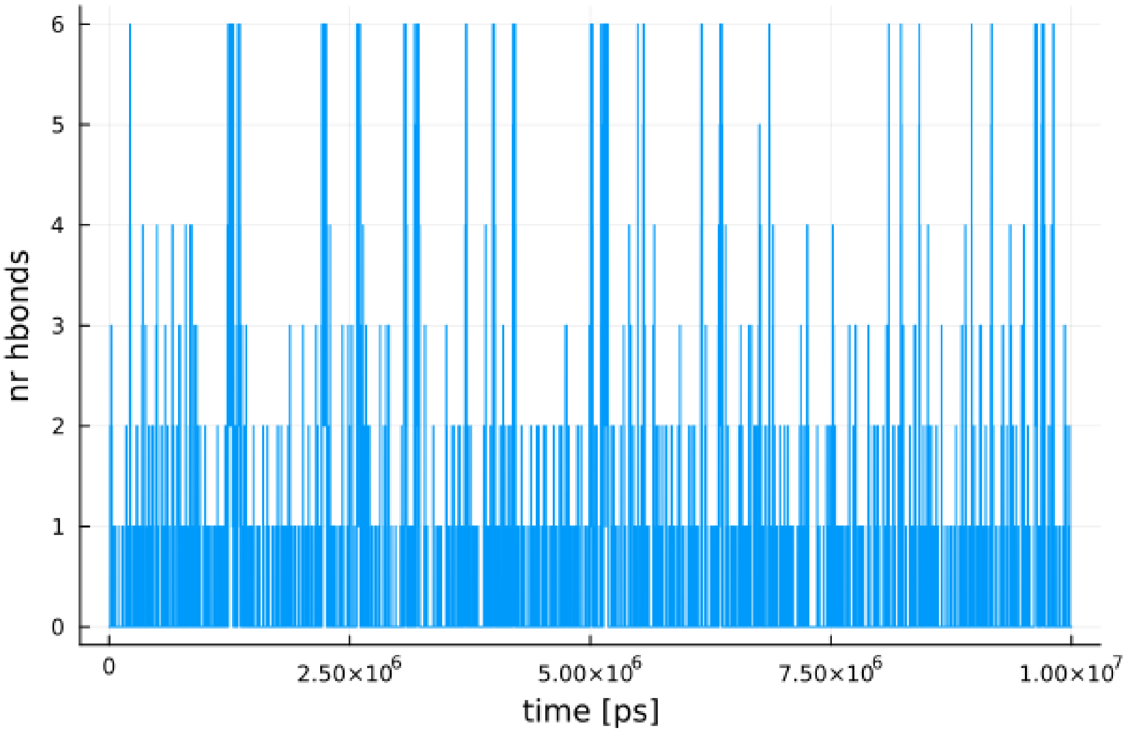

Figure S22: Timeseries showing the evolution over time of the number of active alpha-helix hydrogen bonds for Ala8e.

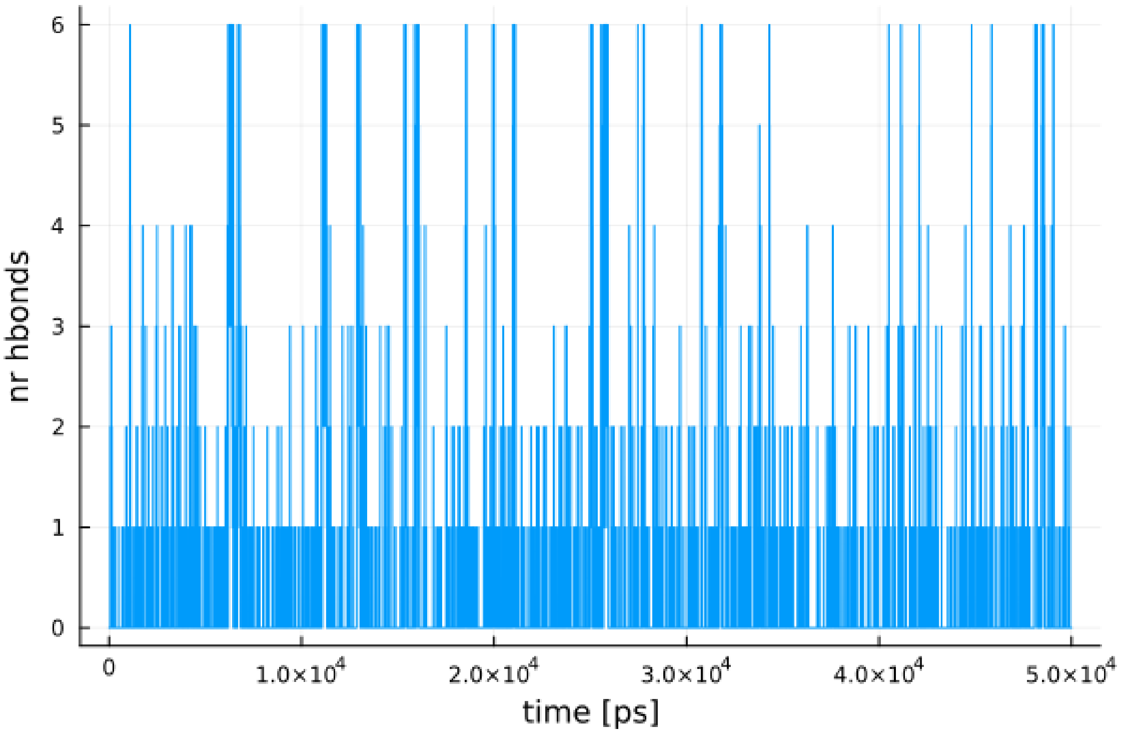

Figure S23: Timeseries showing the evolution over time of the number of active alpha-helix hydrogen bonds for Ala8e. The data was smoothed by eliminating cases where the number of hydrogen bonds changed, and then reverted in the next frame.

Simultanous folding pathways for x_i calculations

Several folding pathways occurring simultaneously have been exemplified in the main paper in Fig. 2. Namely, along one folding trajectory of the ALA8 peptide one can discern at least a few folding pathways, see Fig. 2, lower right, as well as Fig. S24. In other words, only for sufficiently high numbers of hydrogen bonds the distribution of end-to-end distances is unimodal and the correct end-to-end distance is straightforwardly defined as the maximum of this distribution, see Fig. S24, the left sub-figure. However, for small numbers of hydrogen bonds the distribution usually has several maxima, which are associated with various folding pathways, and merging them together does not appear to shift significantly an overall center of mass, see Fig. S24, the right sub-figure.

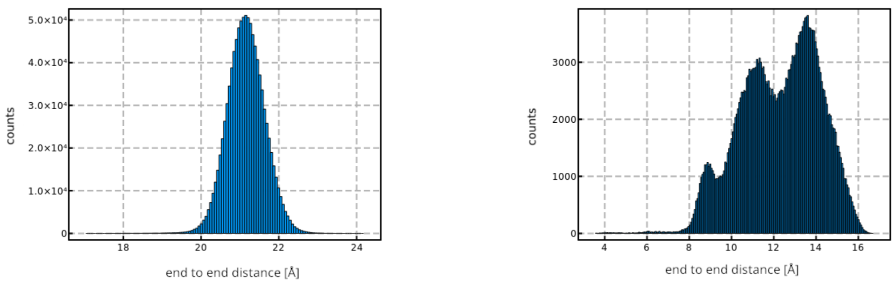

Figure S24: Left: Ala15h 2M of proline, distribution of end-to-end distances for structures with 12 hydrogen bonds formed. Right: Ala8h 2M of proline, distribution of end-to-end distances for structures with 3 hydrogen bonds formed.

Additional data for diffusion constant calculations: mean square deviation

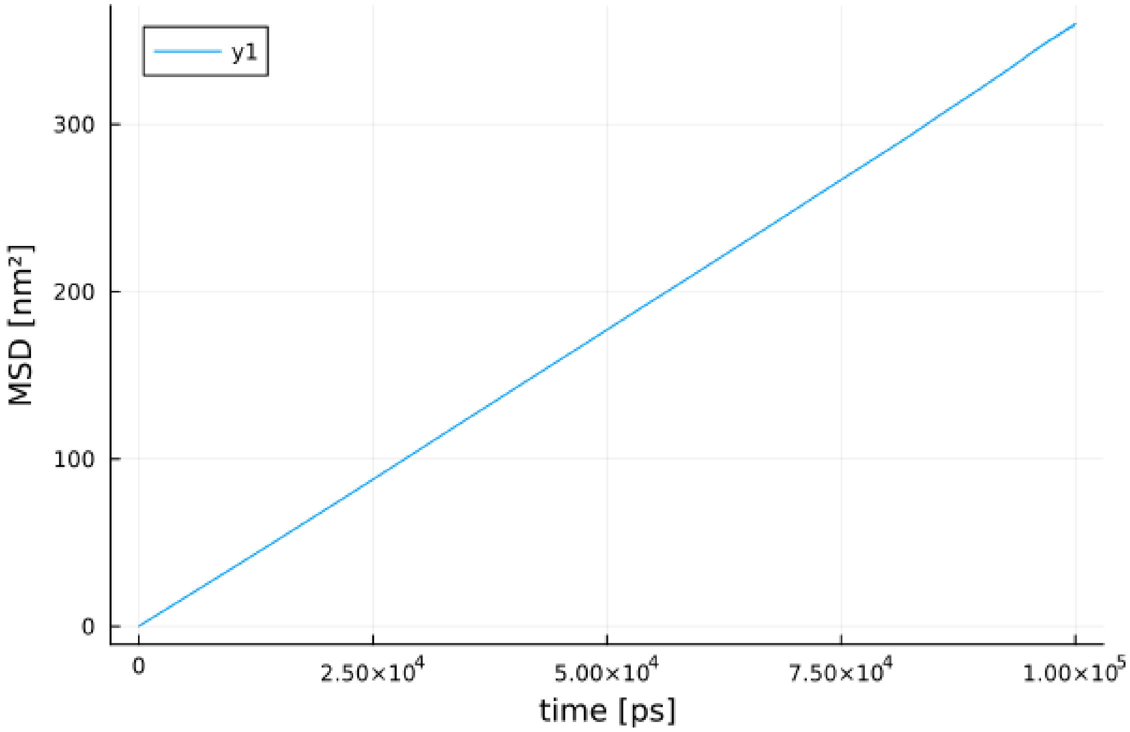

Figure S25: Example mean square deviation trace for KR1"e" in 5M of proline. It increases linearly in accordance with theory.

References

(1) Hungerland, J.; Frederiksen, A.; Gerhards, L.; Solov'yov, I. A. Studying folding ↔ unfolding dynamics of solvated alanine polypeptides using molecular dynamics. *The European Physical Journal D* **2022**, *76*, 154 (13 pages).

(2) Sigworth, F.; Sine, S. Data transformations for improved display and fitting of single-channel dwell time histograms. *Biophysical Journal* **1987**, *52*, 1047–1054.

(3) Szoszkiewicz, R.; Ainavarapu, S. R. K.; Wiita, A. P.; Perez-Jimenez, R.; Sanchez-

Ruiz, J. M.; Fernandez, J. M. Dwell Time Analysis of a Single-Molecule Mechanochemical Reaction. *Langmuir* **2008**, *24*, 1356–1364, Publisher: American Chemical Society.

NON_AVERAGED PARTIAL DATA for each starting configurations ("e" means exte

kB, [m2 kg s-2 K-1]	T, [K]	D_wo, [cm^2/s]
1,38E-23	300	5,6635E-05

name	τ_long [ps]	τ_short [ps]	helicality_hb
ala5h-5m	25233	1760	0,044783333
ala5e-5m	41669,04	2014,68	0,073496667
ala8h-5m	157000	1849	0,121001667
ala8e-5m	393659,79	5072,54	0,19982
ala15h-5m	637448,79	42935,09	0,82735
ala15e-5m	223000	19610	0,78261
aaqaa3h-5m	591365,8743	89053,51883	0,775403077
aaqaa3e-5m	555537,3513	15149,45248	0,200073636
kr1h-5m	2160130,337	176504,0081	0,358303
kr1e-5m	1228630,29	69271,7213	0,142773
ala5h-2m	2955	995	0,03696
ala5e-2m	2910	434	0,02948
ala8h-2m	138159	8487	0,102826667
ala8e-2m	69187	4790	0,098255
ala15h-2m	608964	8387	0,534128462
ala15e-2m	964613	7555	0,383304615
aaqaa3h-2m	210275,6106	16452,9437	0,251302308
aaqaa3e-2m	203569,4732	14634,37555	0,24065
kr1h-2m	285538	3119	0,155803
kr1e-2m	181018	2339	0,086916
ala5h-0m	2160	483	0,02623
ala5e-0m	2230	320	0,03148
ala8h-0m	11100	319	0,059855
ala8e-0m	14500	613	0,066761667
ala15h-0m	119000	681	0,219373846
ala15e-0m	43100	1760	0,278183846
aaqaa3h-0m	117000	4330	0,235456154
aaqaa3e-0m	112000	1680	0,19844
kr1h-0m	34600	4970	0,05006
kr1e-0m	53000	5060	0,062407
ala21h-0m	306000	3250	0,590073158
ala21e-0m	419000	6970	0,585232105

ended; "h" means helical starting configurations)

helicality_ppa	k_unfold [1/µs]	k_fold [1/µs]	Δx [Å]
0,10024	37,85585014	1,774792269	4,205284757
0,122166	22,23481351	1,763819533	4,231241986
0,15855875	5,598715499	0,770711253	4,010090531
0,22406875	2,032668869	0,507595658	4,331386876
0,77259	0,270845286	1,297908182	4,150948137
0,729645333	0,974843049	3,509461883	8,324154333
0,73323	0,379793513	1,311207005	2,886732738
0,225734286	1,439914637	0,360144347	7,741613827
0,36851	0,297064019	0,16587101	3,216965607
0,180945	0,697709479	0,116205014	3,689814955
0,10129	325,9018613	12,50761421	4,156755569
0,09534	333,5120275	10,13058419	4,239095296
0,15245	6,493774082	0,744263252	4,425330117
0,14816375	13,03344559	1,420136731	4,926682755
0,526935333	0,765023119	0,877110078	1,953467984
0,396289333	0,639318965	0,397366214	4,010665217
0,284970667	3,560554123	1,195109157	0,877359286
0,276873333	3,73017618	1,182151706	3,809678372
0,193639167	2,956513669	0,545647164	3,367346797
0,135803333	5,044161354	0,480151145	3,607038259
0,09037	450,8194444	12,14351852	4,317042994
0,095486	434,3139013	14,11659193	4,294641448
0,1169875	84,69774775	5,392342342	4,194984522
0,1218875	64,36126437	4,604252874	4,487119623
0,254499333	6,559883646	1,843477699	3,013636042
0,305759333	16,74747457	6,454381581	2,443162427
0,269649333	6,534562788	2,012445759	1,902092334
0,237202	7,156785714	1,771785714	3,151704198
0,0992275	27,45491329	1,446820809	3,105075348
0,131088333	17,69043396	1,177490566	1,828622081
0,583458095	1,339630203	1,928343653	3,725076565
0,577766667	0,98989951	1,396735335	3,568700678

γ_l [ng/s]	γ_g [µg/s]	τ_dwell [ps]	D [cm^2/s]	D_wo/D
13,74940184	13,815	37,34494206	0,000005424	10,442
13,22080818	14,157	35,90922145	5,34825E-06	10,589
27,55363336	38,020	74,83880776	5,79113E-06	9,780
32,1956985	54,356	87,44718562	5,70563E-06	9,926
109,6441851	107,275	297,8061	5,89225E-06	9,612
118,2050652	7,835	321,0584257	5,54025E-06	10,222
30,51070084	168,778	82,87053998	5,91775E-06	9,570
14,90363297	23,989	40,47996531	6,13525E-06	9,231
26,35997443	376,020	71,59669411	5,01913E-06	11,284
27,53054661	305,404	74,77610153	5,78663E-06	9,787
6,271508365	1,990	17,03413132	2,79934E-05	2,023
6,238463433	2,344	16,94437752	2,90312E-05	1,951
15,47556118	31,675	42,0333875	0,000028857	1,963
15,08324674	13,325	40,96781677	0,000031572	1,794
49,83318518	265,627	135,3526091	3,05365E-05	1,855
49,87019687	105,078	135,4531371	2,83183E-05	2,000
22,53530288	601,361	61,20845036	3,07304E-05	1,843
24,44395505	31,792	66,39256714	2,75041E-05	2,059
18,08645298	79,300	49,12486712	3,05355E-05	1,855
14,69532931	72,613	39,91418885	3,28308E-05	1,725
4,724796741	1,879	12,83308631	5,11012E-05	1,108
4,70363978	1,643	12,77562159	5,26226E-05	1,076
10,327812	4,643	28,05151418	5,13803E-05	1,102
10,4847313	4,788	28,47772488	5,23258E-05	1,082
32,41440107	31,691	88,04120671	5,30789E-05	1,067
31,27742318	14,894	84,95304521	0,000053999	1,049
10,16493279	74,407	27,60911568	5,30747E-05	1,067
10,1158256	29,361	27,47573495	5,31469E-05	1,066
10,16493279	31,257	27,60911568	5,30747E-05	1,067
10,1158256	112,198	27,47573495	5,31469E-05	1,066
39,68489995	37,761	107,7887101	5,34525E-05	1,060
40,856549	56,139	110,9710424	5,31114E-05	1,066

AVERAGED DATA for each peptide

(two starting conformations "e" and "h" were averaged)

path	τ_long [ps]	τ_long [ps] err	τ_short [ps]	τ_short [ps] err
ala5-5m	33451,02	8218,02	1887,34	127,34
ala8-5m	275329,895	118329,895	3460,77	1611,77
ala15-5m	430224,395	207224,395	31272,545	11662,545
aaqaa3-5m	573451,6128	17914,26149	52101,48565	36952,03318
kr1-5m	1694380,314	465750,0233	122887,8647	53616,14337
ala5-2m	2932,5	22,5	714,5	280,5
ala8-2m	103673	34486	6638,5	1848,5
ala15-2m	786788,5	177824,5	7971	416
aaqaa3-2m	206922,5419	3353,06867	15543,65963	909,2840769
kr1-2m	233278	52260	2729	390
ala5-0m	2195	35	401,5	81,5
ala8-0m	12800	1700	466	147
ala15-0m	81050	37950	1220,5	539,5
aaqaa3-0m	114500	2500	3005	1325
kr1-0m	43800	9200	5015	45
ala21-0m	362500	56500	5110	1860

helicality_hb	helicality_hb err	helicality_ppa	helicality_ppa err
0,05914	0,014356667	0,111203	0,010963
0,160410833	0,039409167	0,19131375	0,032755
0,80498	0,02237	0,751117667	0,021472333
0,487738357	0,28766472	0,479482143	0,253747857
0,250538	0,107765	0,2747275	0,0937825
0,03322	0,00374	0,098315	0,002975
0,100540833	0,002285833	0,150306875	0,002143125
0,458716538	0,075411923	0,461612333	0,065323
0,245976154	0,005326154	0,280922	0,004048667
0,1213595	0,0344435	0,16472125	0,028917917
0,028855	0,002625	0,092928	0,002558
0,063308333	0,003453333	0,1194375	0,00245
0,248778846	0,029405	0,280129333	0,02563
0,216948077	0,018508077	0,253425667	0,016223667
0,0562335	0,0061735	0,115157917	0,015930417
0,587652632	0,002420526	0,580612381	0,002845714

k_unfold [1/µs]	k_unfold [1/µs] err	k_fold [1/µs]	k_fold [1/µs] err
30,04533182	7,810518319	1,769305901	0,005486368
3,815692184	1,783023315	0,639153455	0,131557797
0,622844168	0,351998882	2,403685033	1,105776851
0,909854075	0,530060562	0,835675676	0,475531329
0,497386749	0,20032273	0,141038012	0,024832998
329,7069444	3,80508312	11,3190992	1,18851501
9,763609836	3,269835754	1,082199992	0,337936739
0,702171042	0,062852077	0,637238146	0,239871932
3,645365152	0,084811028	1,188630431	0,006478726
4,000337512	1,043823843	0,512899155	0,03274801
442,5666729	8,25277155	13,13005522	0,986536705
74,52950606	10,16824169	4,998297608	0,394044734
11,65367911	5,093795461	4,14892964	2,305451941
6,845674251	0,311111463	1,892115737	0,120330023
22,57267363	4,882239666	1,312155688	0,134665122
1,164764857	0,174865346	1,662539494	0,265804159

Δx [Å]	Δx [Å] err	γ_l [ng/s]	γ_l [ng/s] err
4,218263371	0,012978615	13,48510501	0,264296829
4,170738704	0,160648173	29,87466593	2,321032571
6,237551235	2,086603098	113,9246251	4,28044003
5,314173282	2,427440545	22,7071669	7,803533938
3,453390281	0,236424674	26,94526052	0,58528609
4,197925433	0,041169864	6,254985899	0,016522466
4,676006436	0,250676319	15,27940396	0,196157221
2,982066601	1,028598617	49,85169102	0,018505842
2,343518829	1,466159543	23,48962896	0,954326087
3,487192528	0,119845731	16,39089114	1,695561831
4,305842221	0,011200773	4,714218261	0,01057848
4,341052073	0,14606755	10,40627165	0,07845965
2,728399235	0,285236808	31,84591212	0,568488944
2,526898266	0,624805932	10,14037919	0,024553595
2,466848714	0,638226633	10,14037919	0,024553595
3,646888621	0,078187943	40,27072447	0,585824527

γ_g [μg/s]	γ_g [μg/s] err	τ_dwell [ps]	τ_dwell [ps] err	D [cm^2/s]
13,98615535	0,170728751	36,62708176	0,717860304	5,38613E-06
46,18803287	8,167654777	81,14299669	6,30418893	5,74838E-06
57,55515148	49,72005773	309,4322629	11,62616285	5,71625E-06
96,38367049	72,3945173	61,67525264	21,19528734	6,0265E-06
340,7120893	35,30813539	73,18639782	1,589703711	5,40288E-06
2,167220483	0,177111961	16,98925442	0,0448769	2,85123E-05
22,49998603	9,174549682	41,50060214	0,532785364	3,02145E-05
185,3521221	80,27453824	135,4028731	0,050263976	2,94274E-05
316,5764886	284,7848654	63,80050875	2,592058391	2,91173E-05
75,95649205	3,343499072	44,51952798	4,605339131	3,16831E-05
1,760988196	0,118462165	12,80435395	0,028732358	5,18619E-05
4,715152827	0,072443278	28,26461953	0,213105348	5,18531E-05
23,29284381	8,398594424	86,49712596	1,544080748	5,3539E-05
51,88393318	22,52323324	27,54242532	0,066690361	5,31108E-05
71,72753582	40,47038925	27,54242532	0,066690361	5,31108E-05
46,95001729	9,189030021	109,3798763	1,591166167	5,3282E-05

D [cm^2/s] err
3,7875E-08
4,275E-08
0,000000176
1,0875E-07
3,8375E-07
5,189E-07
1,3575E-06
1,10913E-06
1,61313E-06
1,14763E-06
7,607E-07
4,7275E-07
4,6005E-07
3,61E-08
3,61E-08
1,7055E-07
